# Synthesis and Anticancer Potential of New Benzimidazole Theranostic

**DOI:** 10.1002/open.202500263

**Published:** 2025-07-15

**Authors:** Sahani Sandalima Uthumange, Muhammad Azri Faiz bin Abdul Zaki, Keng Yoon Yeong

**Affiliations:** ^1^ School of Science Monash University Malaysia Jalan Lagoon Selatan 47500 Subang Jaya Selangor Malaysia

**Keywords:** anticancer, benzimidazole, fluorescent, selectivity, sirtuin

## Abstract

A series of novel benzimidazole analogs is designed, synthesized, and screened against a panel of selected cancer cell lines, including H103 (oral squamous cell carcinoma, OSCC), H314 (OSCC), and HCT116 (colorectal carcinoma). Structural characterization of the compounds is successfully confirmed using nuclear magnetic resonance spectroscopy (^1^H and ^13^C) and liquid chromatography‐mass spectrometry. Within the series, compound **V7** emerged as a promising anticancer candidate, displaying broad‐spectrum activity with high selectivity toward the tested cancer cell lines (half‐maximal inhibitory concentration, IC_50_: H103 = 11.64 μM, H314 = 16.68 μM, HCT11 = 13.30 μM). Furthermore, the observed sirtuin 2 (SIRT2) inhibitory activity of **V7** suggests a potential link to its anticancer effects. Molecular docking analysis reveals the importance of a hydroxyl group at the *ortho* position of the 2‐phenyl ring in rendering SIRT2 inhibitory activity. Notably, the high autofluorescent properties of **V7** (molar absorptivity *ε* = 34,477 M^−1^ cm^−1^, quantum yield Φ = 26%, and Stokes shift Δ*λ *= 166 nm) indicate potential for further development as a theranostic agent for cancer.

## Graphical abstract

Recent advances have highlighted benzimidazole's importance in anticancer drug discovery. A novel series of benzimidazole analogs is designed and synthesized, with **V7** showing broad anticancer activity and selectivity, possibly linked to SIRT2 inhibition.

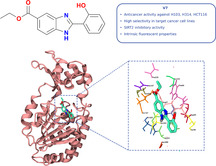

## 1 Introduction

Recent advancements in drug innovation and medicinal chemistry have underscored the significance of the benzimidazole scaffold.^[^ ^1^, ^2^, ^3^ ^]^ This planar, heterocyclic moiety acts as an interesting building block for the synthesis of numerous biologically active compounds that have been shown to possess a wide spectrum of unique properties.^[^ ^4^, ^5^, ^6^, ^7^ ^]^ Diverse synthetic strategies have been developed wherein the benzimidazole scaffold can be modified to produce a variety of functionalized molecules.^[^ ^8^, ^9^, ^10^ ^]^

Given its broad pharmacological potential and promising role in drug development, the benzimidazole pharmacophore has attracted considerable attention for its ability to address complex diseases such as cancer.^[^ ^11^ ^]^ Recent work by Messaoudi et al. described the anticancer activity of novel benzoyl aryl benzimidazole derivatives against MCF7 breast cancer, HepG2 hepatocellular carcinoma, and HCT116 colorectal carcinoma cell lines, with IC_50_ values ranging from 0.06 to 0.5 μg mL^−1^.^[^ ^12^ ^]^ Molecular docking studies indicated that the compounds effectively bind within the catalytic site of caspase‐3. A 2024 study by Abbade et al. reported the synthesis of a series of alkylsulfonyl 1* H*‐benzo[*d*]imidazole derivatives, with lead compounds demonstrating notable activity against MCF7 cells (IC_50 _= 4.7–10.9 μM), potentially through inhibition of the antiapoptotic protein Bcl‐2.^[^ ^13^ ^]^ In the same year, a new 1,2,4‐triazole benzimidazole was shown to exhibit cytotoxic effects against the A549 lung carcinoma cells (IC_50 _= 4.56 μM), as well as significant DNA topoisomerase I inhibitory activities.^[^ ^14^ ^]^ Laxmikeshav et al. also presented a series of benzimidazole‐based carboxamide derivatives as tubulin polymerization inhibitors, with highly selective cytotoxicity against SK‐Mel‐28 skin melanoma cells (IC_50_ value = 2.55–17.89 μM).^[^ ^15^ ^]^ Collectively, these studies highlight the benzimidazole scaffold as a versatile framework for the rational design of novel anticancer agents acting through multiple molecular targets and pathways.

Among the new discoveries, previous work by our group has unveiled the potential of benzimidazoles as sirtuin inhibitors.^[^ ^16^, ^17^, ^18^ ^]^ Sirtuins are nicotinamide adenine dinucleotide (NAD^+^)‐dependent class III histone deacetylase enzymes that are generally involved in regulating the cellular response to oxidative and metabolic stress.^[^ ^19^ ^]^ The mammalian sirtuin family comprises seven members (SIRT1‐7) that differ in their localization, activities, and functions.^[^ ^20^ ^]^ Of the seven, SIRT1‐3 are the most extensively studied. Several sirtuins are found to be overexpressed in various tumor types and implicated in promoting tumorigenesis.^[^ ^21^, ^22^ ^]^ Sirtuins are also known to regulate numerous downstream targets, including forkhead box class O (FOXO),^[^ ^23^ ^]^ nuclear factor‐ĸb (NK‐ĸB),^[^ ^24^ ^]^ and p53^[^ ^25^ ^]^ proteins, which are closely associated with the pathogenesis of cancer development.

**Figure 1:**
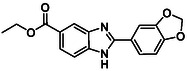
Compound **BZD9Q1.**

In our previous study, we highlighted compound **BZD9Q1** (**Figure** **1**) as a pan‐SIRT1‐3 inhibitor with strong antiproliferative activity against the H103 oral squamous cell carcinoma (OSCC) cell line.^[^ ^16^ ^]^ To support the design and discovery of novel bioactive compounds with potential anticancer properties, this study aims to further refine the active scaffold by introducing modifications to the benzodioxole moiety and subsequently evaluating the anticancer potential of the resulting derivatives.

With such consideration, we present a novel series of 2‐substituted benzimidazole analogs bearing various hydroxy and methoxy substitutions on the phenyl ring. The antiproliferative activity of the compounds was evaluated against a panel of cancer cell lines, including H103 (OSCC), H314 (OSCC), and HCT116 (colorectal carcinoma). Selectivity for cancerous cells was assessed by comparing the antiproliferative effects on HEK293, a noncancerous human embryonic kidney cell line. Furthermore, the SIRT2 inhibitory activity of the compounds was reported, and molecular docking studies were performed to analyze the structure–activity relationship of the novel series.

## 2 Experimental Section

### 2.1 Chemistry

All chemicals and solvents were purchased from Sigma‐Aldrich (USA), Merck Chemicals (Germany), Alfa Aesar (USA), and Acros Organics (USA). ^1^H and ^13^C nuclear magnetic resonance (NMR) were performed on the Bruker Avance NEO 300 NMR Spectrometer. Mass spectra were recorded using a Waters Xevo TQ‐XS Triple Quadrupole Mass Spectrometer.

### 2.2 Synthetic Procedure of the Compounds

**Figure 2:**
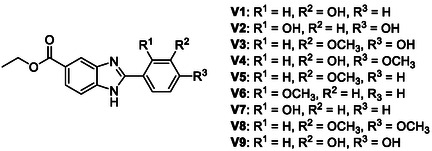
General structure of the synthesized benzimidazoles.

A series of nine 2‐substituted benzimidazole analogs (**Figure** **2**) were synthesized using a method adapted from literature.^[^ ^16^ ^]^

4‐Fluoro‐3‐nitrobenzoic acid (3.0 g, 16.2 mmol) was dissolved in ethanol (30 mL), followed by the addition of concentrated sulfuric acid (6.0 mL). The mixture was stirred under reflux at 125 °C for 7 h. Reaction progress was monitored using thin‐layer chromatography (TLC) (ethyl acetate/hexane, 1:4 *v*/*v*). Upon completion, ice was added to induce precipitation. The solid was collected by vacuum filtration, washed with cold water, and dried to yield Ethyl 4‐fluoro‐3‐nitrobenzoate (Intermediate I) as a white solid (3.27 g, 15.3 mmol, 94.2%).

Ethyl 4‐fluoro‐3‐nitrobenzoate (3.27 g, 15.3 mmol) was dissolved in ethanol (30 mL), and ammonium hydroxide (7 mL) was added. The reaction was stirred at room temperature overnight. Completion was confirmed by TLC (ethyl acetate/hexane, 1:3 *v*/*v*). The mixture was then heated to 50 °C with an open cap to remove excess ammonia. After cooling, the resulting solid was isolated by vacuum filtration, washed with cold ethanol, and dried to afford Ethyl 4‐(2‐substituted‐amino)‐3‐nitrobenzoate (Intermediate II) as a yellow solid (2.39 g, 11.4 mmol, 74.2%).

Ethyl 4‐(2‐substituted‐amino)‐3‐nitrobenzoate (0.42 g, 2.0 mmol), ammonium formate (0.37 g, 6.0 mmol), and Pd/C (50 mg) were suspended in ethanol (10 mL) and heated at 70 °C under reflux for 30–45 min, until the solution turned from green to colorless. The mixture was centrifuged (1500 rpm, 5 min) to remove Pd/C, and the supernatant was filtered. Ethanol was removed under reduced pressure, and the residue was redissolved in ethyl acetate (20 mL), washed with water (3 × 10 mL), and dried over anhydrous sodium sulfate. Solvent evaporation yielded Ethyl 4‐(2‐substituted‐amino)‐3‐aminobenzoate (Intermediate III) as an orange solid (0.30 g, 1.65 mmol, 82.5%).

*N*‐substituted benzaldehyde sodium bisulfite adducts were prepared by dissolving the corresponding benzaldehydes (5 mmol) in ethanol (10 mL) and adding the solution dropwise to sodium metabisulfite (7.5 mmol) dissolved in water (3 mL). The mixture was stirred at room temperature for 1 h, then stored at 4 °C overnight. The resulting precipitate was collected by vacuum filtration and washed with cold ethanol.

Intermediate III (Ethyl 4‐(2‐substituted‐amino)‐3‐aminobenzoate, 1.0 eq) and intermediate IV (1.5 eq) were dissolved in DMF (5 mL) and stirred at 90 °C under reflux for 24–48 h. Reaction completion was monitored by TLC (chloroform/methanol, 9:1 *v*/*v*). The mixture was diluted with ethyl acetate (25 mL), washed with water (3 × 10 mL), and dried over anhydrous sodium sulfate. After solvent removal, the crude product was purified by recrystallization from ethyl acetate to afford the final compounds.

### 2.3 Cell Proliferation Assay

The H103 and H314 OSCC cell lines were obtained from the European Collection of Cell Cultures (ECACC). The HCT116 and HEK293 cell lines were obtained from the American Type Culture Collection (Rockville, MD).

All cell lines were maintained in high‐glucose Dulbecco's Modified Eagle Media (DMEM) (Sigma‐Aldrich, Buchs, Switzerland). The culture media was supplemented with 10% fetal bovine serum (FBS) (TICO, Amsterdam, Netherlands) and 1% penicillin‐streptomycin. Prior to the cell‐based assay, cell lines were incubated at 37 °C in a 5% CO_2_ humidified atmosphere

Cells were seeded in 96‐well plates at a density of 5 × 103 per well. After 24 h of seeding, the cells were treated with 50 μM of the compounds of interest (final concentration 0.25% DMSO) and allowed to adhere for 72 h at 37 °C in a 5% CO_2_ humidified atmosphere. The 3‐(4,5‐dimethylthiazol‐2‐yl)‐2,5‐diphenyltetrazolium bromide (MTT) assay was conducted to examine the cell cytotoxicity profiles of the synthesized compounds. Briefly, the treatment media was discarded, and 0.5 mg mL^−1^ of MTT was added to each well. The plates were incubated for another 4 h at 37 °C in a 5% CO_2_ humidified atmosphere. Following the incubation, the medium‐MTT solution was removed, and 100 μL of DMSO was added to dissolve the formazan crystals. The absorbance was measured at 570 nm, with the reference wavelength at 620 nm, using the Tecan Infinite 200 Pro. The experiment was carried out in triplicate.

### 2.4 SIRT2 Enzymatic Assay

The SIRT2 inhibitory activity of the compounds was evaluated using the SIRT2 Direct Fluorescent Screening Assay Kit (Cayman Chemicals, Ann Arbor, C/N: 700,280) according to the manufacturer's instructions. The SIRT2 peptide substrate comprised amino acids 317–320 of human p53 conjugated to 7‐dimethylamino‐4‐methylcoumarin (Gln‐Pro‐Lys‐Lys(ε‐acetyl)‐AMC). The peptide substrate (125 μM), cofactor NAD^+^ (2 mM), test compounds (50 μM), and SIRT2 human recombinant were incubated on a shaker for 45 min at 37 °C. Subsequently, 50 μl of developer solution (6 μg μl^−1^ SIRT2 developer, 2 mM nicotinamide) was added. The mixture was incubated for a further 30 min at room temperature. AGK‐2 (SIRT2 selective inhibitor) was used as the standard control. The fluorescence was measured with excitation and emission wavelengths of 355 and 460 nm, respectively, using the Tecan Infinite 200 Pro. The percentage inhibition was determined as the ratio of absorbance under each experimental condition to that of the control.

### 2.5 Molecular Docking

The crystal structure of human SIRT2 (PDB code: 3ZGV) was obtained from the Protein Data Bank. Prior to conducting the docking simulation, the receptor and ligands were structurally optimized. Following the removal of co‐crystallized water molecules, hydrogen atoms were incorporated into the protein structure. Ligands were energy minimized using the MM2 force field in Chem3D Pro 22.2.0. Docking was carried out using Autodock 4.2.6. A control docking experiment with ADPr and 3ZGV produced a low RMSD value (1.23 Å), indicating that a reliable docking protocol was employed. The top‐ranked pose for each ligand was retained and analyzed using VMD 1.9.3 molecular graphics software (3D visualization) and Schrodinger (2D visualization).

### 2.6 Physicochemical Properties

#### 2.6.1 Molar Extinction Coefficient

A series of dilutions of **V7** (dissolved in DMSO) was prepared. The absorbance of the solutions at 326 nm was recorded on the UviLine 9400 spectrophotometer using a quartz cuvette (10 × 10 mm). A calibration curve of absorbance against concentration was generated. The molar extinction coefficient was calculated from the gradient of the line of best fit, in accordance with Beer–Lambert's law.

#### 2.6.2 Quantum Yield

A series of dilutions of **V7** (dissolved in DMSO) was prepared. The fluorescence emission peak at each concentration was determined using the Tecan Infinite 200 Pro microplate reader (λex = 326 nm and λem = 492 nm). The absorbance peak was plotted against the fluorescence emission peak and compared to that of a quinine sulfate standard, which has a known quantum yield.

The quantum yield of **V7** was subsequently calculated using the following equation

$$\Phi =\Phi_{R}\left(\frac{Grad}{Grad_{R}}\right)\left(\frac{n^{2}}{n_{r}^{2}}\right)$$

Φ = Quantum yield

$\Phi_{R}$ = Quantum yield of reference

Grad = gradient of the absorbance versus concentration plot for the sample

$Grad_{R}$ = gradient of absorbance versus concentration plot for reference

n = refractive index of the sample

$n_{r}$ = refractive index of reference

The quinine sulfate reference has a quantum yield of 54.6% when dissolved in 0.5 M H_2_SO_4_.^[^ ^26^ ^]^ The refractive index of 0.5 M H_2_SO_4_ is 1.346, while the refractive index of DMSO is 1.479.^[^ ^27^ ^]^

## 3 Results and Discussion

### 3.1 Synthesis of Compounds

**Figure 3:**
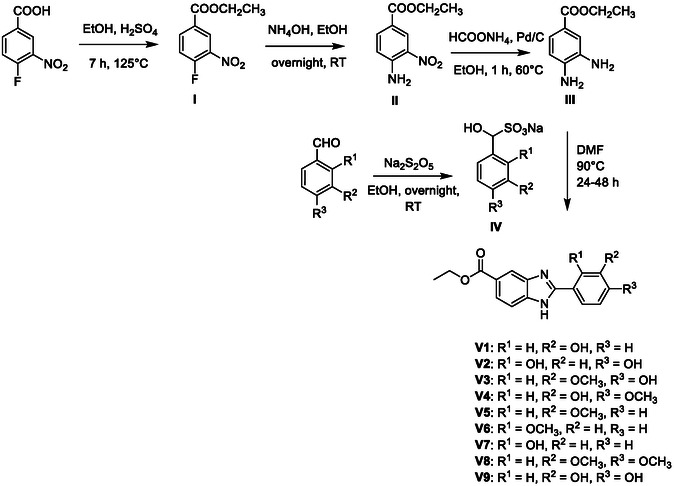
Synthesis of compounds V1‐9 presented in this study.

The benzimidazole derivatives were synthesized following the scheme illustrated in **Figure** **3**. Initially, 4‐fluoro‐3‐nitrobenzoic acid was esterified with ethanol and catalytic sulfuric acid to afford ethyl 4‐fluoro‐3‐nitrobenzoate (intermediate I). A nucleophilic aromatic substitution with ammonium hydroxide produced ethyl 4‐(2‐substituted‐amino)‐3‐nitrobenzoate (intermediate II). Reduction of the nitro group using ammonium formate and Pd/C afforded the corresponding diamine (intermediate III). To obtain the final compounds **V1‐9** (**Table** **1**), various aromatic aldehydes were converted to their bisulfite adducts by reaction with sodium metabisulfite in ethanol. These adducts were subsequently condensed with intermediate III to yield the target benzimidazole derivatives in 24.5–86.8% yields.

**Table 1:** Substituent patterns (R^1^, R^2^, R^3^) and percentage yields of synthesized compounds **V1‐V9**.

Compound	R^1^	R^2^	R^3^	Percentage yield (%)
**V1**	H	OH	H	40.6
**V2**	OH	H	OH	83.4
**V3**	H	OCH_3_	OH	27.3
**V4**	H	OH	OCH_3_	62.9
**V5**	H	OCH_3_	H	86.8
**V6**	OCH_3_	H	H	53.1
**V7**	OH	H	H	59.0
**V8**	H	OCH_3_	OCH_3_	24.5
**V9**	H	OH	OH	76

**Figure 4:**
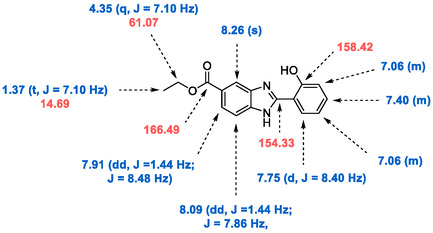
Selected ^1^H and ^13^C NMR chemical shifts of **V7**.

Since the biological activities of 4‐hydroxyphenyl and 4‐methoxyphenyl derivatives have been previously reported,^[^ ^17^ ^]^ they were excluded from the present study. The synthesized compounds were characterized using ^1^H NMR, ^13^C NMR, and LC‐MS analyses (Supplementary Information), and the results were in full agreement with the proposed structures. ^1^H and ^13^C NMR assignments for **V7** are presented as a representative example for the other compounds in the series (**Figure** **4**). The ^1^H NMR spectrum of **V7** displays a characteristic quintet at 4.35 ppm, corresponding to the methylene protons adjacent to the ester oxygen (–CH_2_–). Methyl protons (–CH_3_) appear as a triplet around 1.37 ppm, while aromatic protons fall within the 7.06–8.26 ppm region. In compounds featuring –OCH_3_ substitutions, a singlet near 3.85 ppm is attributed to the methoxy protons. The ^13^C NMR spectrum of **V7** includes signals at 14.69 and 61.07 ppm, assigned to the methyl (–CH_3_) and methylene (–CH_2_–) carbons of the ethyl ester group, respectively. The carbonyl (C=O) carbon resonates at 166.49 ppm, and the phenolic (C–OH) carbon at 158.42 ppm. Consistent patterns were observed across all compounds (see Supplementary Information). Mass spectrometry further confirmed compound **V7**, showing a molecular ion peak at m/z 282.83 [M + H]^+^. The purity of the synthesized benzimidazole derivatives (**V1‐V9**) was confirmed by ultra‐performance liquid chromatography (UPLC), with all compounds exhibiting purity levels above 96%.

### 3.2 Anticancer Activities

The anticancer activities of the synthesized benzimidazole derivatives were examined through a preliminary screening against two OSCC (H103 and H314) and one colorectal carcinoma cell line (HCT116) after 72 h of incubation at 50 μM concentration (Figure 4).

All derivatives (**V1**–**V9**) exhibited substantial cytotoxic effects on the H103 cell line. One‐way ANOVA followed by Tukey's post‐hoc test revealed highly significant differences between all treatment groups and the control (0.25% DMSO), with *p *< 0.001. As shown in Figure 4, the derivatives were generally less effective against the H314 cell line compared to H103. All compounds, except **V1**, **V4**, and **V8**, showed statistically significant cytotoxicity (*p* < 0.05) in H314 cells, with **V5**, **V7**, and **V9** demonstrating highly significant effects (*p* < 0.001). In the HCT116 cell line, all compounds except **V1** produced statistically significant cytotoxicity (*p *< 0.001). Among the tested compounds, **V5**, **V7**, and **V9** displayed the best overall antiproliferative activity (**Table** **2**).

**Table 2:** IC_50_ values and selectivity indices of selected compounds across various cell lines (HEK293, H103, H314, and HCT116).

Compound	H103		H314		HCT116		HEK293
	IC_50_ [μM] ± SEM	Selectivity Index (SI)	IC_50_ [μM] ± SEM	Selectivity Index (SI)	IC_50_ [μM] ± SEM	Selectivity Index (SI)	IC_50_ [μM] ± SEM
**BZD9Q1**	5.83^[^ ^16^ ^]^	3.15	>100	NDa)	11.48 ± 2.30	1.60	18.39 ± 2.95
**V5**	22.80 ± 3.84	2.00	31.65 ± 1.27	1.44	11.18 ± 3.14	4.08	45.58 ± 2.18
**V7**	11.64 ± 1.04	3.81	16.68 ± 1.13	2.66	13.30 ± 2.52	3.34	44.37 ± 3.03
**V9**	>50	NDa)	37.79 ± 1.81	1.37	16.56 ± 3.52	3.13	51.79 ± 5.65
**Cisplatin**	1.85 ± 0.42	1.91	15.08 ± 1.05	0.23	8.24 ± 0.86	0.43	3.54 ± 0.18

a) ND = Not Determined.

For the H103 cell line, **V7** demonstrated the most potent inhibitory effect on cellular proliferation, with an IC_50_ value of 11.64 μM. In comparison, our previous study reported an IC_50_ of 5.83 μM for **BZD9Q1** in H103 cells.^[^ ^16^ ^]^ However, further analysis revealed that **BZD9Q1** exhibited substantially lower potency against the slower‐growing H314 cell line (IC_50_ > 100 μM), highlighting a distinct selectivity in its mode of action. In contrast, the novel compound **V7** consistently showed inhibitory effects in H314 cells, with an IC_50_ of 16.68 μM. Furthermore, the tested series of compounds displayed similar levels of activity in HCT116 cells.

The potency of **V7** in the H314 cell line was found to be comparable to that of the clinically validated cisplatin (IC_50 _= 15.08 μM). However, the sensitivity to cisplatin was 8‐fold greater in H103, which suggests the presence of resistance mechanisms in H314 cells. These findings echo a recent study by Khoo et al., wherein a differential response to cisplatin was reported in the H103 and H314 cell lines, as indicated by IC_50_ values of 15 μM and 200 μM (24 h treatment), respectively.^[^ ^28^ ^]^ The authors deduced that cisplatin resistance in the H314 cell line is likely mediated by an increased production of extracellular vesicles, which promote drug efflux.

The selected compounds also showed significantly lower cytotoxicity against non‐cancerous HEK293 cells compared to cisplatin (Table 2). Notably, compound **V7** demonstrated better selectivity than cisplatin across all three tested cancer cell lines, with both compounds displaying comparable potency in the H314 and HCT116. The novel compounds also demonstrated lower cytotoxicity in HEK293 cells compared to the previously identified **BZD9Q1**, with **V7** exhibiting superior selectivity indices across all cell lines. Collectively, the high anticancer activity and selectivity demonstrated by **V7** positions this compound as the lead candidate in the series.

The ability of a cytotoxic agent to selectively target desired cells is a highly desirable characteristic, as it suggests a potential for reduced side effects.^[^ ^29^ ^]^ Cisplatin's tendency to induce adverse reactions has previously been attributed to its limited cytoselectivity.^[^ ^30^, ^31^ ^]^ Additionally, over 90% of drug candidates failed to be marketed due to toxicity drawbacks.^[^ ^32^ ^]^ Thus, the differential toxicity exhibited by the series is highly significant and bodes well for the development of a compound with a wide therapeutic index.

### 3.3 SIRT2 Enzymatic Activities

As our previous work and others have implicated SIRT2 inhibition in anticancer effects,^[^ ^16^, ^17^, ^18^, ^33^, ^34^ ^]^ the in vitro SIRT2 inhibitory activity of the synthesized compounds was evaluated using the SIRT2 Direct Fluorescent Screening Assay Kit (Cayman Chemicals, Ann Arbor, C/N: 700,280), according to the manufacturer's protocol.

Based on the preliminary screening at 50 μM, the majority of the compounds displayed moderate to poor SIRT2 inhibitory activity (**Table** **3**). Among the series, compounds **V2** and **V7** were the most active against SIRT2, giving a complete inhibition of enzymatic activity. Further studies determined the IC_50_ values of **V2** and **V7** as 10.8 μM and 6.4 μM, respectively. Our previous results showed that AGK2 produced an IC_50_ of 8.34 μM,^[^ ^16^ ^]^ which is comparable to that of compounds **V2** and **V7**.

**Table 3:** Percentage inhibition of SIRT2 by compounds **V1‐9** (50 μM) and AGK2 (10 μM).

Compound	Percentage SIRT2 inhibition (%)
**V1**	25.4 ± 11.8
**V2**	>95 (Complete inhibition)
**V3**	61.9 ± 12.0
**V4**	NIa)
**V5**	NIa)
**V6**	11.8 ± 11.4
**V7**	>95 (Complete inhibition)
**V8**	25.5 ± 12.9
**V9**	39.3 ± 7.3
**AGK2 (positive control)**	62.0 ± 6.0

a) NI = No inhibition

Considering the presence of ortho‐hydroxy groups in both compounds **V2** and **V7**, it can be postulated that having a hydrogen bond‐donating group in the ortho position is vital for establishing key interactions within SIRT2's active site. In contrast, the ortho‐methoxy substitution in **V6** resulted in reduced SIRT2 inhibitory activity. Methoxy‐containing compounds generally showed weaker SIRT2 inhibitory activity, except for **V3**, which also contains a *para*‐hydroxy group like in **V2**. Notably, compounds bearing para‐hydroxy substituents displayed moderate to high SIRT2 inhibition, while para‐methoxy groups were associated with minimal or no activity. Substitution at the meta position appeared to have little influence on SIRT2 inhibition.

Given that sirtuins have been strongly linked to cancer progression,^[^ ^21^, ^22^, ^34^ ^]^ the SIRT2 inhibitory activity of **V7** (SIRT2 IC_50_ = 6.4 μM) may have contributed to its pronounced cytotoxic effects. The role of SIRT2 in colorectal cancer is complex, with evidence pointing to both tumor‐promoting and tumor‐suppressing functions.^[^ ^35^, ^36^ ^]^ However, the detailed association between SIRT2 inhibition and the regression of OSCC has not been previously discussed. As the acetylation status of p53^[^ ^37^ ^]^ and *α*‐tubulin^[^ ^38^ ^]^ is directly modulated by the activity of SIRT2 enzymes, the cytotoxicity of compound **V7** may be attributed to its action on these specific cellular targets. Prior work by our group has shown that treatment with compound **BZD9Q1** significantly increased the level of acetylated α‐tubulin in H103 cells.^[^ ^16^ ^]^ *α*‐Tubulin is a key protein involved in microtubule dynamics and the regulation of cell division. The increased acetylation of *α*‐tubulin is associated with high microtubule stability, which confers resistance to depolymerization and disassembly.^[^ ^39^, ^40^ ^]^ As the stability of microtubules must be transient for the continuous change of organization during the cell cycle, higher microtubule stability as a result of hyperacetylated α‐tubulin can disrupt the process of cell division and ultimately induce cell death.^[^ ^41^, ^42^ ^]^

### 3.4 Molecular Docking

**Figure 5:**
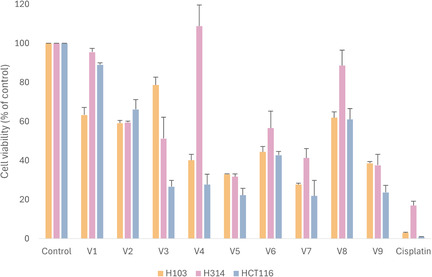
Antiproliferative effect of synthesized compounds against H103, H314, and HCT166 cancer cells after 72 h of incubation at 50 μM concentration. Error bars represent the standard error of mean (SEM) across three biological replicates (*n* = 3). One‐way ANOVA followed by Tukey post‐hoc analysis was conducted to determine the significant differences between the control group and treatment groups.

To analyze the structure–activity relationship of the novel series, molecular docking analysis into the active site of human SIRT2 (PDB entry code: 3ZGV, X‐ray resolution = 2.30 Å) was performed using Autodock 4.2.6 along with AutoDockTools (Scripps Research Institute, La Jolla, CA).^[^ ^43^ ^]^ The compounds of interest were selected based on their dual criteria of SIRT2 inhibition and anticancer activity. Unlike **V7**, although compound **V2** had promising SIRT2 inhibitory potential, its cytotoxicity in the cancer cell lines was suboptimal (see **Figure** **5**). Bearing a di‐hydroxy substituent, **V2**'s increased hydrophilicity (ClogP = 1.75 for **V2** vs 2.29 for **V7**) likely indicates slower cellular uptake, which can in turn also limit its impact on SIRT2.^[^ ^44^, ^45^ ^]^ Alternatively, **V4** and **V5** showed no SIRT2 inhibition, while **V6** exhibited the weakest inhibitory effect (see Table 3). Nonetheless, these compounds demonstrated moderate anticancer activity, suggesting alternative mechanisms of action. As shown in Table 3, **V1** and **V8** exhibited the subsequent lowest levels of SIRT2 inhibition. These compounds also produced a weak antiproliferative effect in the tested cell lines, suggesting a potential correlation with their SIRT2 inhibitory activity. Interestingly, both **V1** and **V8** lack an ortho‐hydroxy substituent, unlike **V7** and **V2**, suggesting a possible role of this group in conferring activity within the context of the investigated compound scaffold. To evaluate the hypothesized SAR, **V7**, **V1**, and **V8** were selected for molecular docking analysis. The receptor and compounds of interest were optimized prior to the docking process in Autodock 4.2.6, which was carried out following the standard procedure of the software.

**Figure 6:**
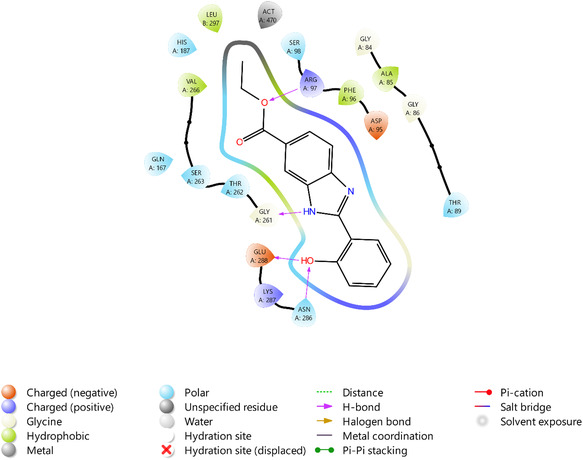
Molecular interactions observed between **V7** and SIRT2.

Docking analysis results revealed that **V7** occupies the adenosine binding pocket (A pocket) within the active site (binding energy = −7.84 kcal mol^−1^). Hydrogen bond formation was evident with residues ARG97, GLY261, GLU288, and ASN286 (**Figure** **6**). Notably, the interactions involving GLU288 and ASN286 occurred with the ortho‐hydroxy group of **V7**. These findings are relatively consistent with the hydrogen bonding reported for the SIRT2‐ADPR complex.^[^ ^43^ ^]^ Similar predictions have also been made for the SIRT2 inhibitor salermide.^[^ ^46^ ^]^ Alterations to these key amino acids make crucial contributions to the inhibitory mechanism.^[^ ^47^, ^48^ ^]^ The stabilization of the binding complex was also facilitated by polar interactions with HIS187, GLN167, SER263, THR262, SER98, and THR89. Additionally, hydrophobic van der Waals interactions were observed between **V7** and residues VAL266, LEU297, ALA85, and PHE96.

**Figure 7:**
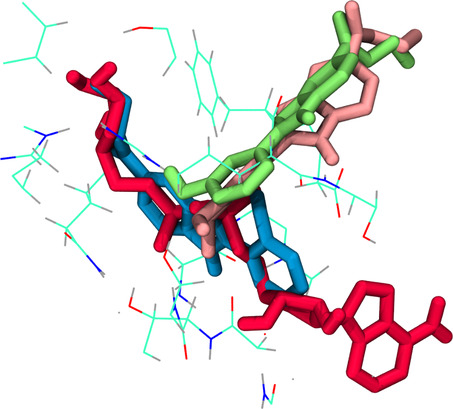
**V7** (blue), ADPr (red), **V8** (pink), and **V1** (green) in the active site of SIRT2.

The overlap of **V7** and ADPr implies that the compound likely inhibits SIRT2 activity by preventing the binding of cofactor NAD^+^ (**Figure** **7**). Comparatively, **V8** and **V1** weaker binding affinities, measuring at −4.96 kcal mol^−1^ and −5.24 kcal mol^−1^, respectively. This suggests that the formed complexes exhibit reduced stability, indicative of weaker interactions between the enzyme and the compounds. As depicted in Figure 7, both **V8** and **V1** adopt a binding mode distinct from **V7**, orienting themselves away from the ADPr binding site and displaying limited similarity to the SIRT2‐**V7** complex. These observations suggest that the inclusion of an *ortho*‐hydroxy substituent, as seen in **V7** and **V2**, facilitates active site interactions that are crucial to the inhibitory mechanism.

### 3.5 Fluorescence Properties

**Figure 8:**
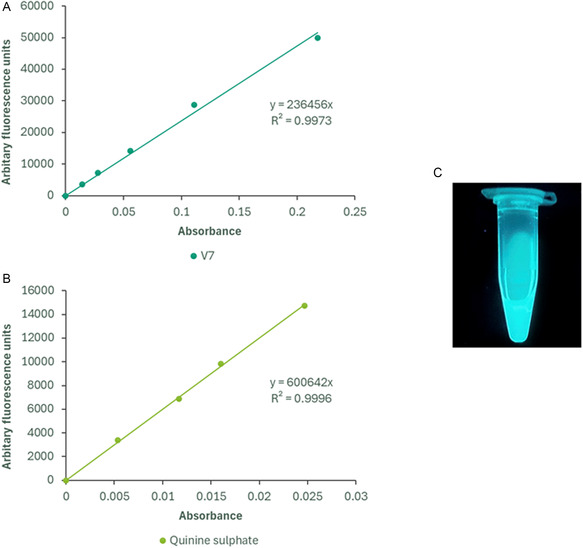
A) Plot of fluorescence intensity versus absorbance for **V7** in DMSO (*λ*_ex_: 326 nm, *λ*_em_: 492 nm). B) Plot of fluorescence intensity versus absorbance for quinine sulfate in 0.5 M H_2_SO_4_ (*λ*_ex_: 348 nm, *λ*_em_: 450 nm). Fluorescence was measured using the Tecan Infinite 200 Pro, and absorbance was recorded with the UviLine 9400 spectrophotometer in a 10 mm quartz cuvette. C) **V7** in DMSO under 365 nm UV light.

The most potent compound of the series, **V7,** was found to have a high degree of autofluorescence when light‐irradiated at *λ*_ex _= 326 nm and *λ*_em _= 492 nm. **V7** demonstrated a high molar extinction coefficient of 34 477 M^−1^ cm^−1^, as well as a quantum yield of 26% (**Figure** **8**). Notably, **V7** also exhibits a substantial Stokes shift of 166 nm, indicating minimal overlap in its absorption and emission spectra. For the practical applications of fluorophores, a Stokes shift greater than 100 nm is a highly desirable, albeit uncommon, characteristic as it reduces the possibility of spectral crosstalk^[^ ^49^ ^]^ and fluorescence self‐quenching.^[^ ^50^, ^51^ ^]^ The autofluorescence properties of **V7** present a compelling avenue for exploring cellular localization and ligand‐receptor interactions, circumventing the recognized disadvantages of fluorescent labeling techniques.^[^ ^52^, ^53^ ^]^ This suggests that **V7** could be developed as a potential theranostic for cancer.

## 4 Conclusion

In conclusion, nine novel benzimidazole derivatives were developed based on a previously reported scaffold. The compounds were found to possess anticancer activity in the tested OSCC and colorectal carcinoma cell lines. Low cytotoxicity against non‐cancerous HEK293 cells highlights a distinct selectivity in their mode of action. Among the series, compound **V7** was identified as the lead candidate due to its high anticancer activity and favorable selectivity profile. Notably, a correlation between in vitro SIRT2 inhibition and cancer cell cytotoxicity was also established, although other possible modes of action against different targets should not be discounted. Investigations into the structure–activity relationship indicated the importance of a hydrogen bond‐donating group at the ortho position of the 2‐phenyl ring in establishing key interactions within SIRT2's active site. Additionally, the strong autofluorescent properties of **V7** offer a promising platform for potential theranostic applications. Collectively, this study exemplifies the utility of the benzimidazole scaffold in the development of novel anticancer agents, providing substantial validation for its potential applications in future drug discovery endeavors.

## Conflict of Interest

The authors declare no conflict of interest.

## Supporting information

Supplementary Material

## Data Availability

The data that support the findings of this study are available from the corresponding author upon reasonable request.
