# Supplementary material for: Synthesis and Anticancer Potential of New Benzimidazole Theranostic
Source: ChemistryOpen. 2025 Jul 15;14(11):e202500263. doi: 10.1002/open.202500263 (PMC12598832; doi:10.1002/open.202500263)
Supplement: Supplementary file 1 — Supplementary Material [file OPEN-14-e202500263-s001.pdf]

Synthesis and Anticancer Potential of New Benzimidazole Theranostic

Sahani Sandalima Uthumange,[a] Muhammad Azri Faiz bin Abdul Zaki [a] and Keng Yoon Yeong [a*]

[a]*School of Science, Monash University (Malaysia Campus), Jalan Lagoon Selatan, Bandar Sunway, 47500, Subang Jaya, Selangor, Malaysia.*

*Corresponding author. Tel.: +603 5514 6102.

Email address: yeong.kengyoon@monash.edu (Keng Yoon Yeong)

Supplementary Information

Table of Contents

Characterization data for the synthesized compounds 1

Supplementary LC-MS data.. 4

Supplementary ^{1}HNMR data 13

Supplementary ^{13}CNMR data.. 22

Supplementary SIRT2 inhibition data.. 38

Supplementary fluorescence studies data 39

Characterization data for the synthesized compounds

Ethyl 2-(3-hydroxyphenyl)-1H-benzo[d]imidazole-5-carboxylate (V1)

Yield: 40.6 %. ^{1}H-NMR (300 MHz, DMSO-d_6, ppm): δ=13.12 (s, 1H, NH), 9.78 (s, 1H, OH), 8.11 (s, 1H, ArH), 7.84 (s,1H, ArH), 7.61 (m, 3H, ArH), 7.37 (t, J = 7.99 Hz, 1H, ArH,), 6.94 (m, 1H, ArH), 4.34 (q, J = 7.10 Hz, 2H, CH_2), 1.36 (t, J = 7.10 Hz, 3H, CH_3). ^{13}C NMR (75 MHz; DMSO-d_6, ppm): δ=166.70, 158.31, 154.33, 131.20, 130.60, 124.13, 123.71, 118.07, 117.96, 114.04, 60.95, 14.72. MS (ESI) *m/z*: $[M+H]^+$ calcd. mass 283.10; mass found 282.83.

Ethyl 2-(2,4-dihydroxyphenyl)-1H-benzo[d]imidazole-5-carboxylate (V2)

Yield: 83.4 %. ^{1}H-NMR (300 MHz, DMSO-d_6, ppm): δ=12.78 (s, 1H, NH), 10.09 (m, 2H, OH), 8.17 (m, 1H, ArH), 7.91 (m, 3H, ArH), 6.48 (dd, J =2.18 Hz; J = 8.61 Hz, 1H, ArH), 6.43 (d, J = 2.29 Hz, 1H, ArH), 4.34 (q, J = 7.09 Hz, 2H, CH_2), 1.36 (t, J = 7.10 Hz, 3H, CH_3). ^{13}C NMR (75 MHz; DMSO-d_6, ppm): δ=166.55, 162.75, 161.82, 160.43, 128.49, 124.26, 123.95, 108.35, 104.71, 103.53, 60.98, 14.69. MS (ESI) *m/z*: $[M+H]^+$ calcd. mass 299.10; mass found 298.86.

Ethyl 2-(4-hydroxy-3-methoxyphenyl)-1H-benzo[d]imidazole-5-carboxylate (V3)

Yield: 27.3 %. ^{1}H-NMR (300 MHz, DMSO-d_6, ppm): δ=13.00 (s, 1H, NH), 9.65 (s, 1H, OH), 7.96 (s, 1H, ArH), 7.82 (d, J = 8.34 Hz, 1H, ArH), 7.76 (d, J = 1.19 Hz, 1H, ArH), 7.65 (dd, J =1.99 Hz; J = 8.23 Hz, 2H, ArH), 6.95 (d, J = 8.26 Hz, 1H, ArH), 7.96 (s, 1H, ArH), 4.34 (q, J = 7.09 Hz, 2H, CH_2), 3.90 (s, 3H, OCH_3), 1.36 (t, J = 7.09 Hz, 3H, CH_3). ^{13}C NMR (75 MHz; DMSO-d_6, ppm): δ=166.77, 149.53, 148.39, 123.73, 121.17, 120.62, 116.25, 111.03, 60.88, 56.18, 14.73. MS (ESI) *m/z*: $[M+H]^+$ calcd. mass 313.11; mass found 312.94.

Ethyl 2-(3-hydroxy-4-methoxyphenyl)-1H-benzo[d]imidazole-5-carboxylate (V4)

Yield: 62.9 %. ^{1}H-NMR (300 MHz, DMSO-d_6): *δ*=13.01 (d, J = 11.23 Hz, 1H, NH), 9.38 (d, J = 5.43 Hz,1H, OH), 8.14 (m, 1H, ArH), 7.82 (m, 1H, ArH), 7.55-7.70 (m, 3H, ArH), 7.12 (dd, J =3.24 Hz; J = 8.36 Hz, H, ArH), 4.34 (q, J = 7.08 Hz, 2H, CH_2), 3.86 (s, 3H, OCH_3), 1.36 (t, J = 7.10 Hz, 3H, CH_3). ^{13}C NMR (75 MHz; DMSO-d_6, ppm): *δ*=166.77, 150.33, 147.24, 123.76, 122.69, 118.83, 114.37, 112.64, 60.90, 56.14, 14.73. MS (ESI) *m/z*: $[M+H]^+$ calcd. mass 313.11; mass found 313.08.

Ethyl 2-(3-methoxyphenyl)-1H-benzo[d]imidazole-5-carboxylate (V5)

Yield: 86.8 %. ^{1}H-NMR (300 MHz, DMSO-d_6, ppm): *δ*=13.24 (s, 1H, NH), 8.20 (m, 1H, ArH), 7.65-7.96 (m, 4H, ArH), 7.50 (t, *J* = 7.89 Hz, 1H, ArH), 7.12 (dd, *J* =3.24 Hz; *J* = 8.36 Hz, 1H, ArH), 7.11 (m, 1H, ArH), 4.35 (q, *J* = 7.09 Hz, 2H, CH_2), 3.88 (s, 3H, OCH_3), 1.36 (t, *J* = 7.10 Hz, 3H, CH_3). ^{13}C NMR (75 MHz; DMSO-d_6, ppm): *δ*=166.68, 160.14, 131.30, 130.70, 124.22, 119.52, 116.91, 112.18, 60.96, 55.80, 14.71. MS (ESI) *m/z*: $[M+H]^+$ calcd. mass 297.12; mass found 296.98.

Ethyl 2-(2-methoxyphenyl)-1H-benzo[d]imidazole-5-carboxylate (V6)

Yield: 53.1 %. ^{1}H-NMR (300 MHz, DMSO-d_6, ppm): *δ*=12.42 (d, *J* = 7.11 Hz, 1H, NH), 8.33 (m, 2H, ArH), 7.85 (m, 1H, ArH), 7.71 (m, 1H, ArH), 7.53 (m, 1H, ArH), 7.28 (dd, *J* =2.86 Hz; *J* = 7.81 Hz, 1H, ArH), 7.15 (m, 1H, ArH), 4.35 (q, *J* = 7.09 Hz, 2H, CH_2), 4.05 (s, 3H, OCH_3), 1.36 (td, *J* = 1.59 Hz; *J* = 7.10 Hz, 3H, CH_3). ^{13}C NMR (75 MHz; DMSO-d_6, ppm): *δ*=166.77, 157.46, 152.39, 134.89, 132.49, 130.43, 123.83, 121.45, 120.55, 118.61, 117.80, 114.41, 112.69, 112.40, 60.90, 56.30, 14.74. MS (ESI) *m/z*: $[M+H]^+$ calcd. mass 297.12; mass found 296.91.

Ethyl 2-(2-hydroxyphenyl)-1H-benzo[d]imidazole-5-carboxylate (V7)

Yield: 59.0 %. ^{1}H-NMR (300 MHz, DMSO-d_6, ppm): δ=8.26 (s, 1H, ArH), 8.09 (dd, J =1.44 Hz; J = 7.86 Hz, 1H, ArH), 7.91 (dd, J = 1.44 Hz; J = 8.48 Hz, 1H, ArH), 7.75 (d, J = 8.40 Hz, 1H, ArH), 7.40 (m, 1H, ArH), 7.06 (m, 2H, ArH), 4.35 (q, J = 7.10 Hz, 2H, CH_2), 1.37 (t, J = 7.10 Hz, 3H, CH_3). ^{13}C NMR (75 MHz; DMSO-d_6, ppm): δ=166.49, 158.42, 154.33, 132.79, 127.26, 124.74, 124.26, 119.80, 117.74, 112.93, 61.07, 14.69. MS (ESI) *m/z*: $[M+H]^+$ calcd. mass 283.10; mass found 282.83.

Ethyl 2-(3,4-dimethoxyphenyl)-1H-benzo[d]imidazole-5-carboxylate (V8)

Yield: 24.5 %. ^{1}H-NMR (300 MHz, DMSO-d_6, ppm): δ=13.09 (s, 1H, NH), 8.17 (m, 1H, ArH), 7.58-7.86 (m, 4H, ArH), 7.16 (d, J = 8.66 Hz, 1H, ArH), 4.34 (q, J = 7.09 Hz, 2H, CH_2), 3.90 (s, 3H, OCH_3), 3.86 (s, 3H, OCH_3), 1.36 (t, J = 7.09 Hz, 3H, CH_3). ^{13}C NMR (75 MHz; DMSO-d_6, ppm): δ=166.81, 154.06, 151.34, 151.19, 149.43, 139.02, 123.88, 123.19, 122.52, 120.31, 120.19, 118.65, 113.00, 112.32, 110.35, 60.94, 60.88, 56.09, 14.72. MS (ESI) *m/z*: $[M+H]^+$ calcd. mass 327.13; mass found 326.94.

Ethyl 2-(3,4-dihydroxyphenyl)-1H-benzo[d]imidazole-5-carboxylate (V9)

Yield: 76.0 %. ^{1}H-NMR (300 MHz, DMSO-d_6, ppm): δ=12.91 (s, 1H, NH), 9.56 (s, 1H, OH), 9.30 (s, 1H, OH), 8.12 (s, 1H, ArH), 7.80 (dd, J =1.53 Hz; J = 8.42 Hz, 1H, ArH), 7.62 (m, 2H, ArH), 7.49 (dd, J =2.11 Hz; J = 8.23 Hz, 1H, ArH), 6.90 (d, J = 8.26 Hz, 1H, ArH), 4.33 (q, J = 7.09 Hz, 2H, CH_2), 1.35 (t, J = 7.09 Hz, 3H, CH_3). ^{13}C NMR (75 MHz; DMSO-d_6, ppm): δ=166.79, 154.96, 148.53, 146.12, 123.61, 123.36, 121.27, 119.13, 116.31, 114.77, 60.87, 14.74. MS (ESI) *m/z*: $[M+H]^+$ calcd. mass 299.10; mass found 298.94.

Supplementary LC-MS data

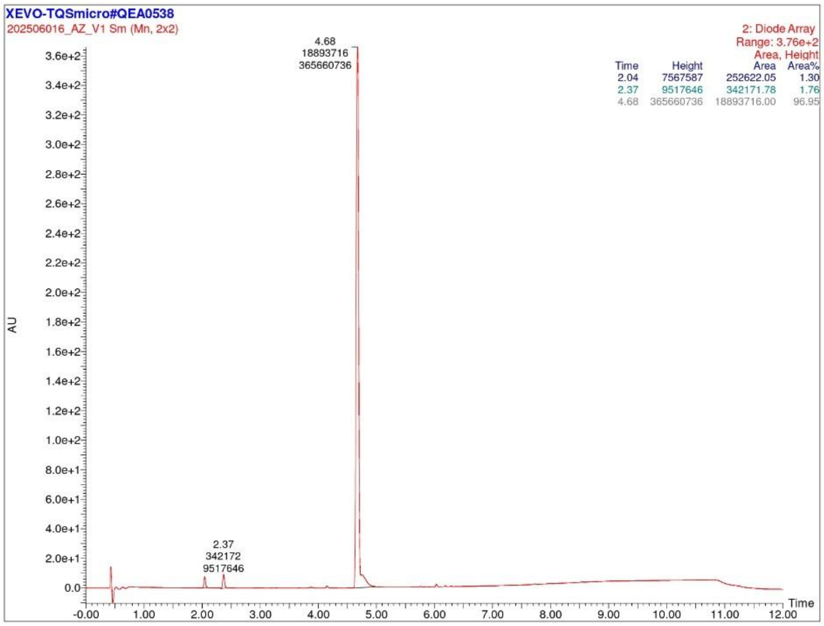

Figure S1: LC-UV chromatogram of compound **V1**

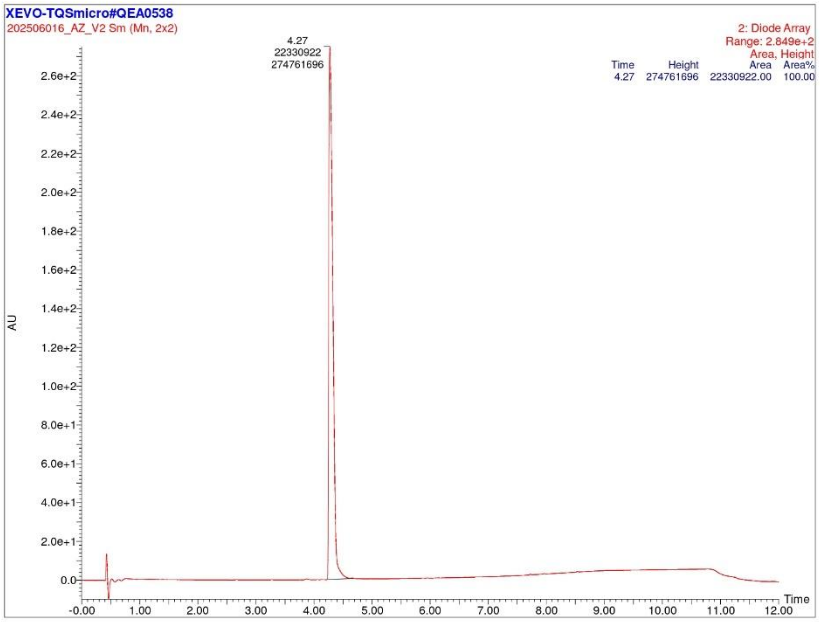

Figure S2: LC-UV chromatogram of compound **V2**

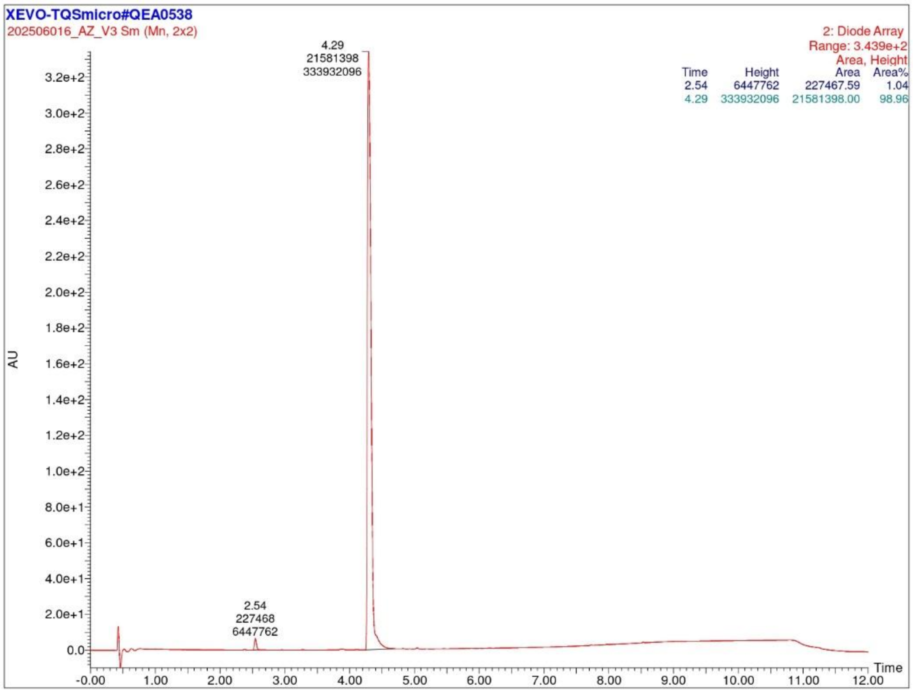

Figure S3: LC-UV chromatogram of compound **V3**

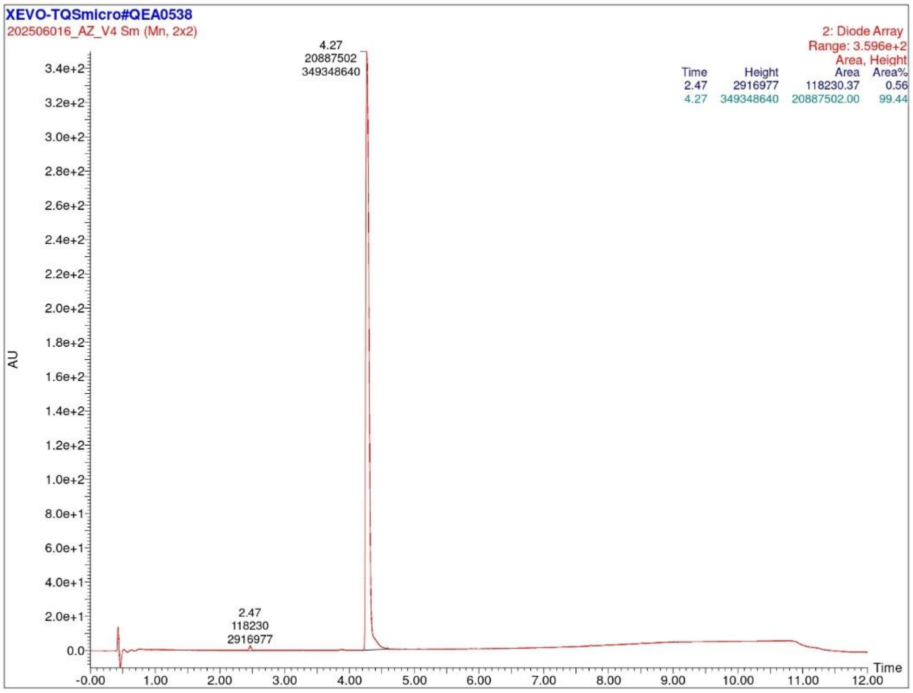

Figure S4: LC-UV chromatogram of compound **V4**

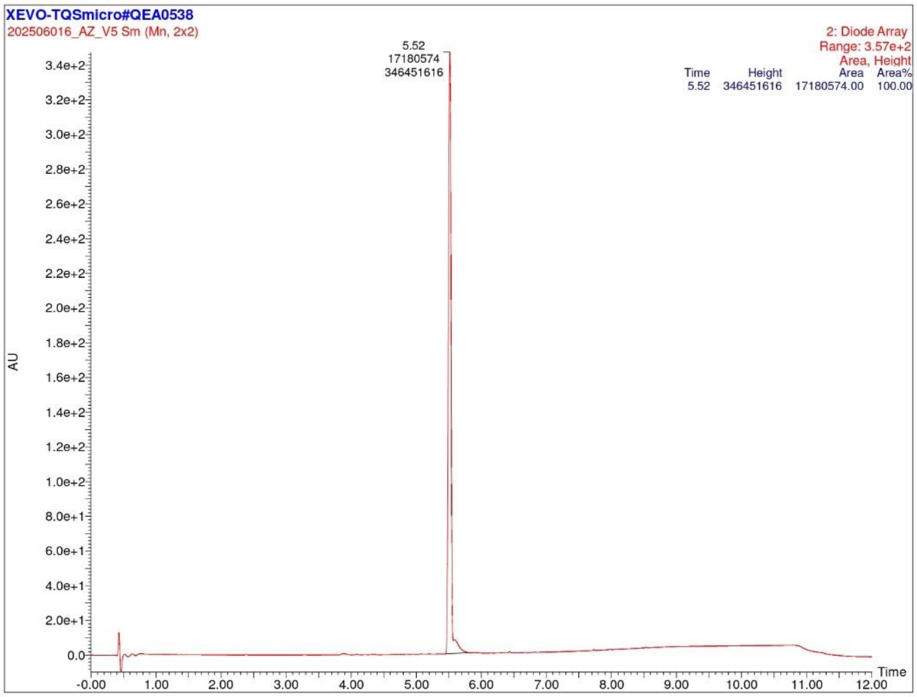

Figure S5: LC-UV chromatogram of compound **V5**

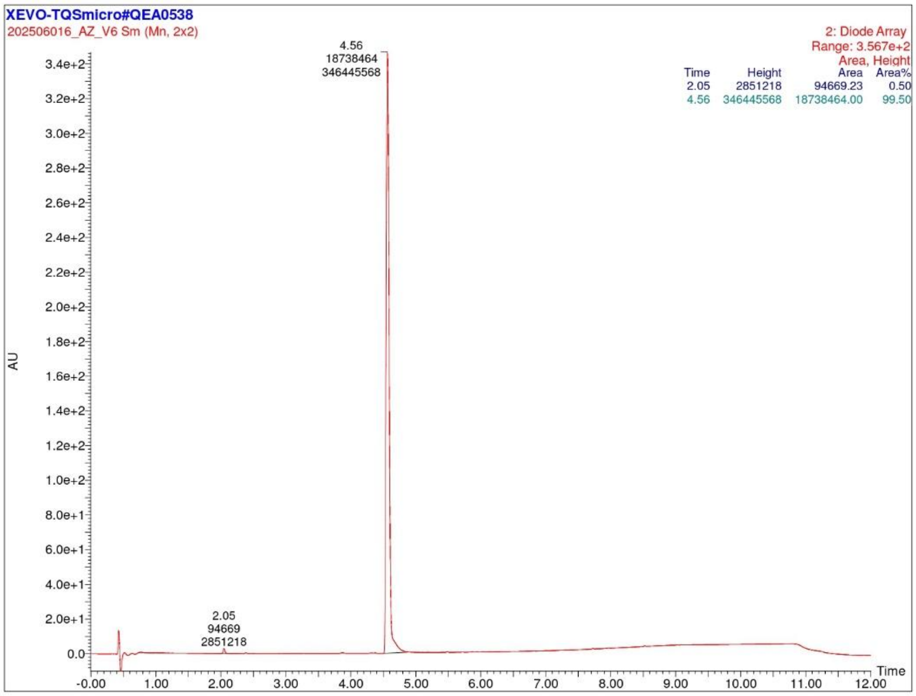

Figure S6: LC-UV chromatogram of compound **V6**

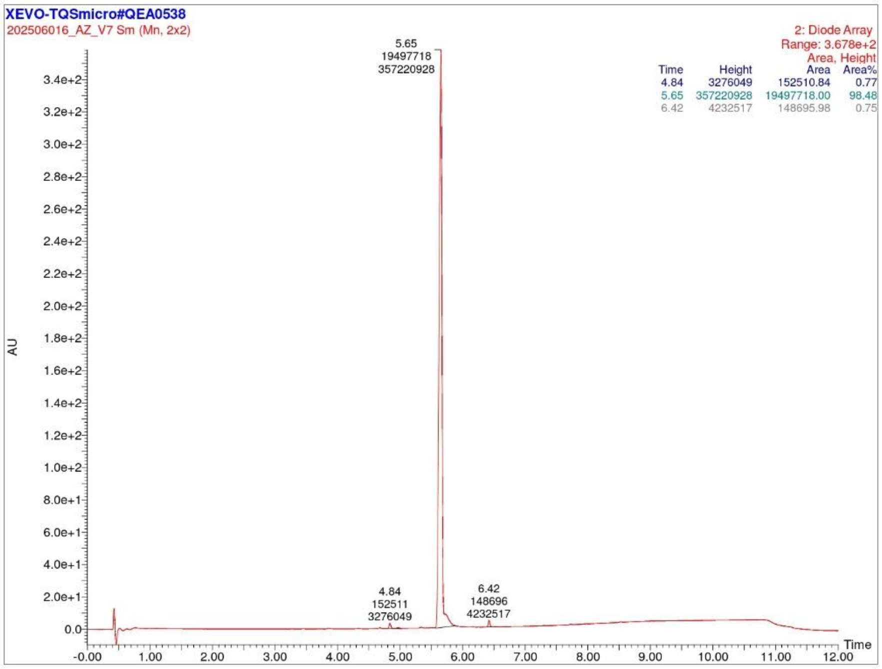

Figure S7: LC-UV chromatogram of compound **V7**

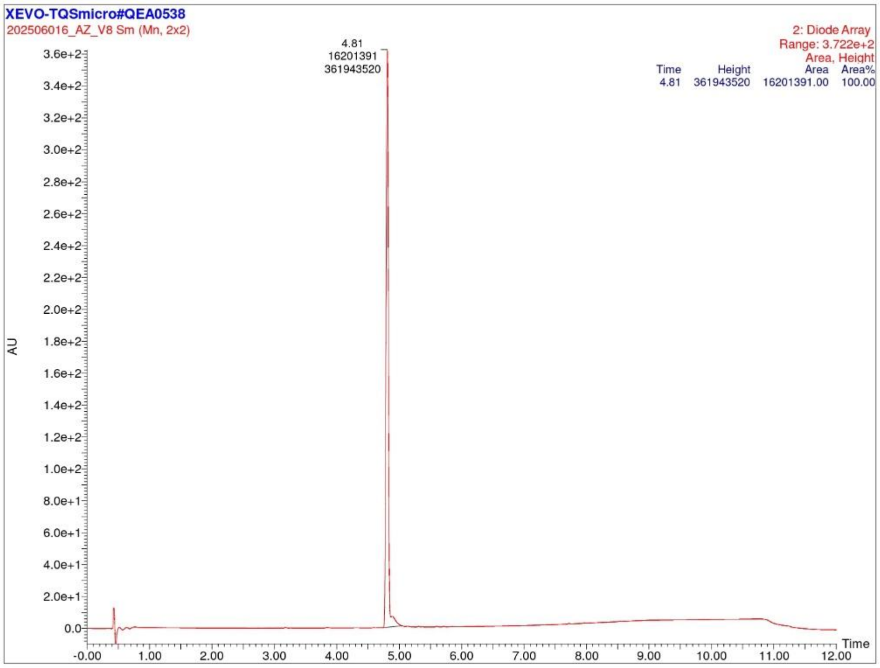

Figure S8: LC-UV chromatogram of compound **V8**

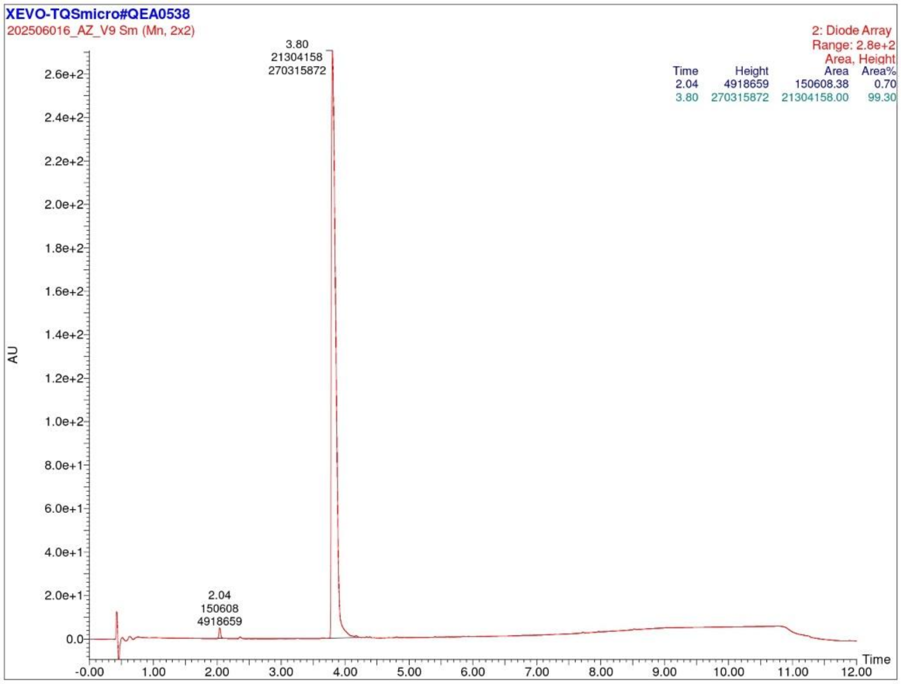

Figure S9: LC-UV chromatogram of compound **V9**

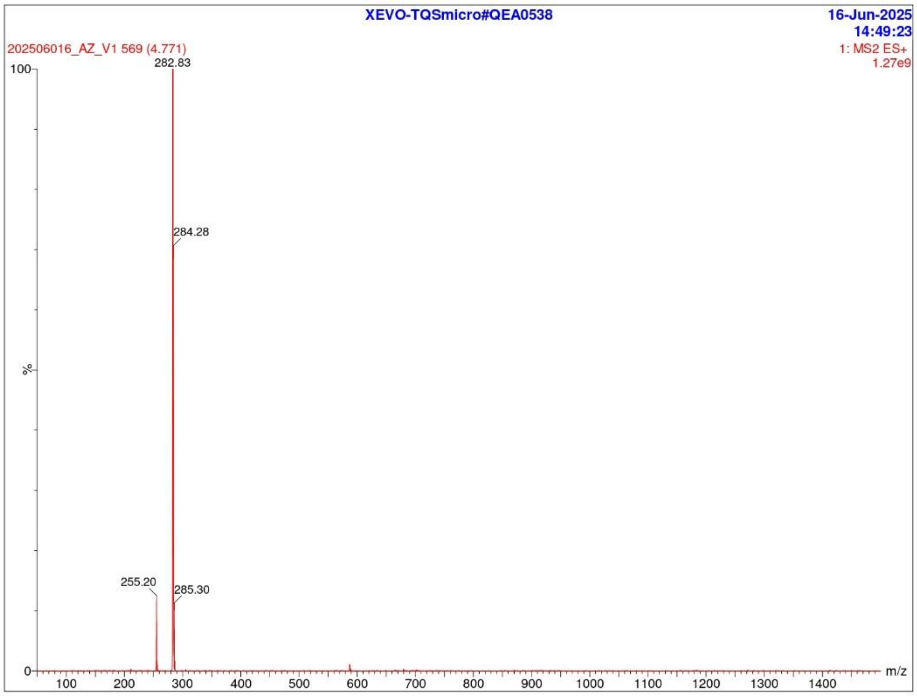

Figure S10: Mass spectrum of compound **V1**

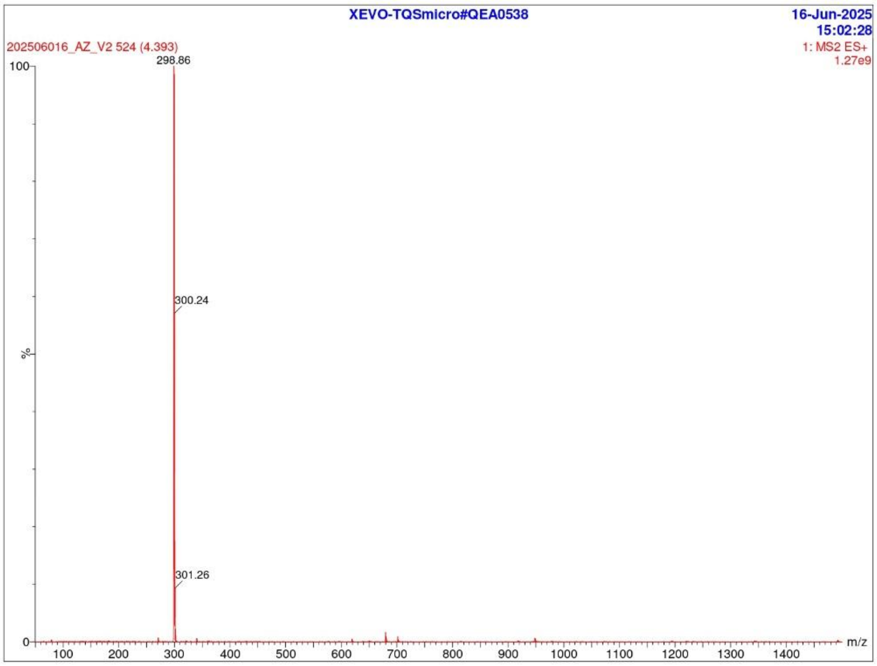

Figure S11: Mass spectrum of compound **V2**

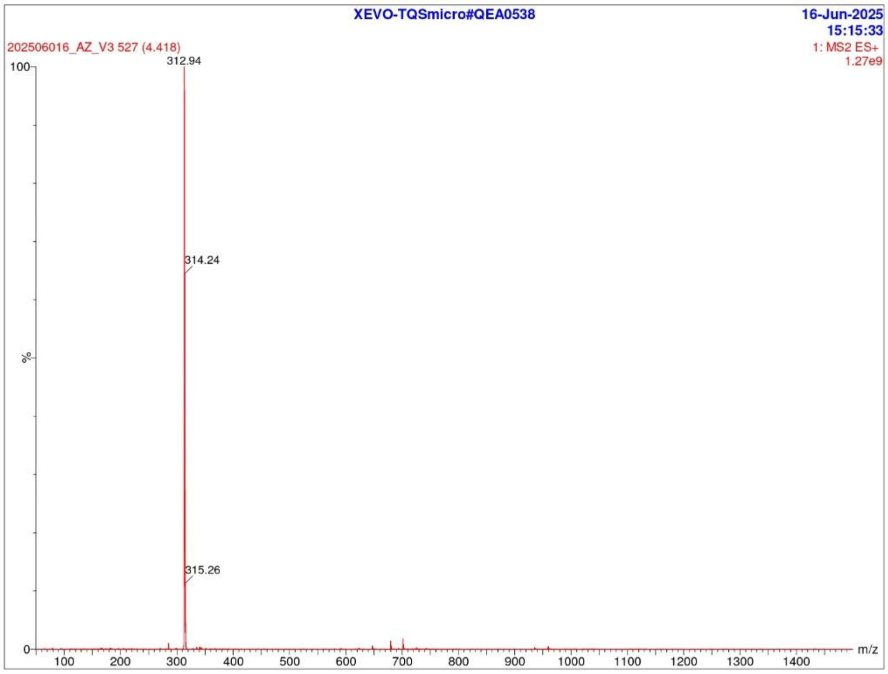

Figure S12: Mass spectrum of compound **V3**

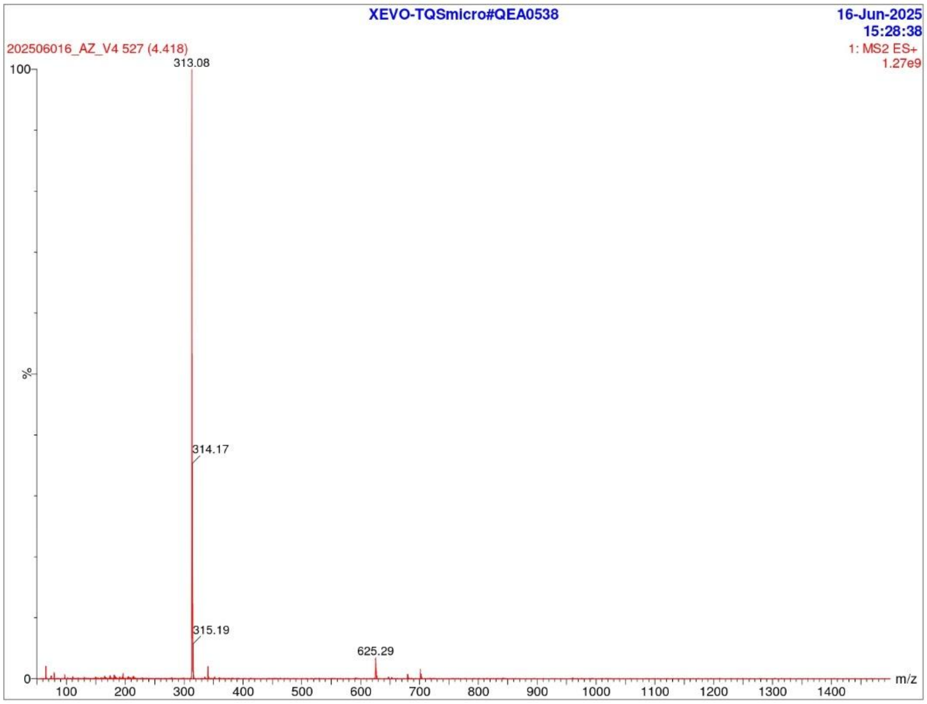

Figure S13: Mass spectrum of compound **V4**

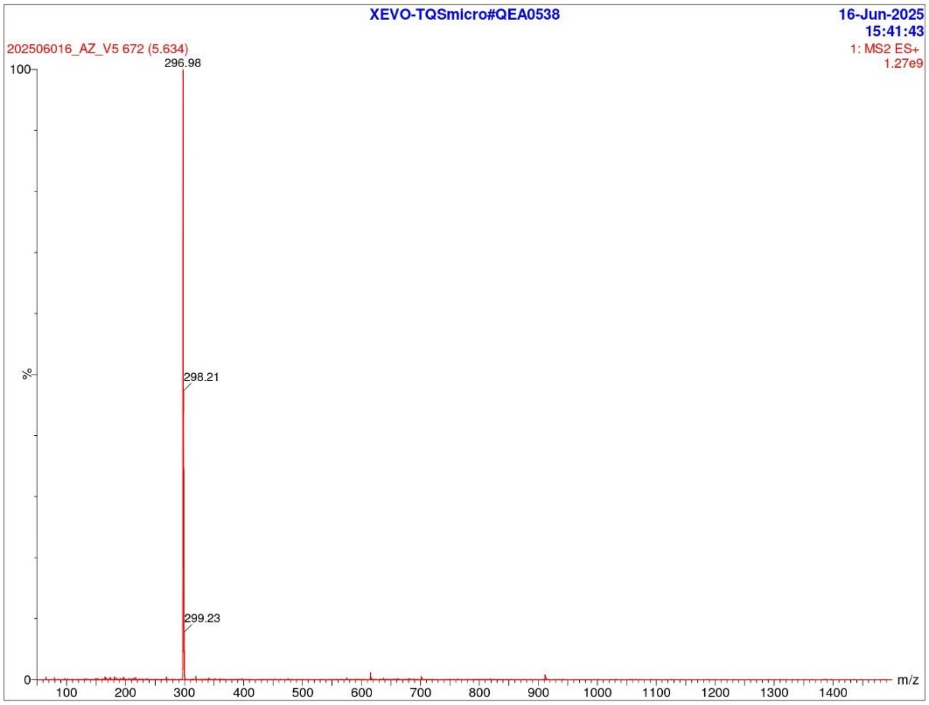

Figure S14: Mass spectrum of compound **V5**

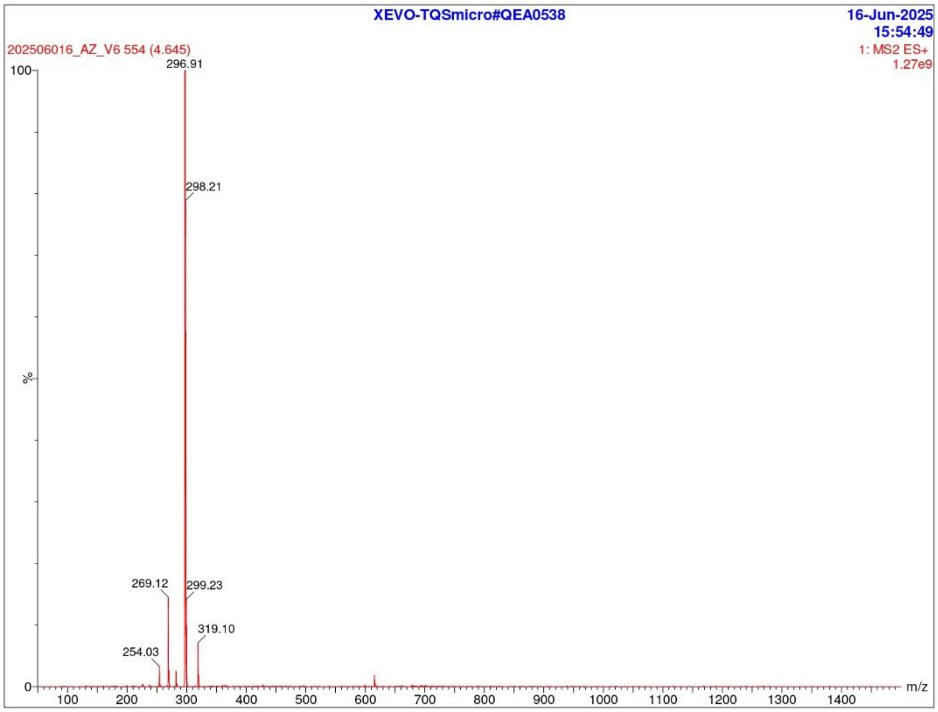

Figure S15: Mass spectrum of compound **V6**

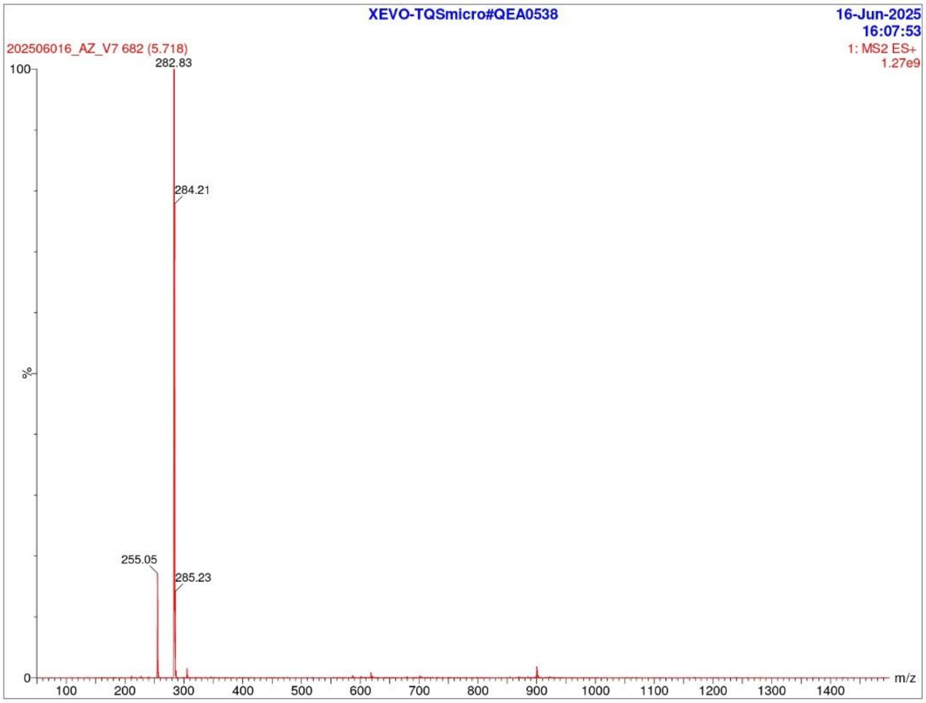

Figure S16: Mass spectrum of compound **V7**

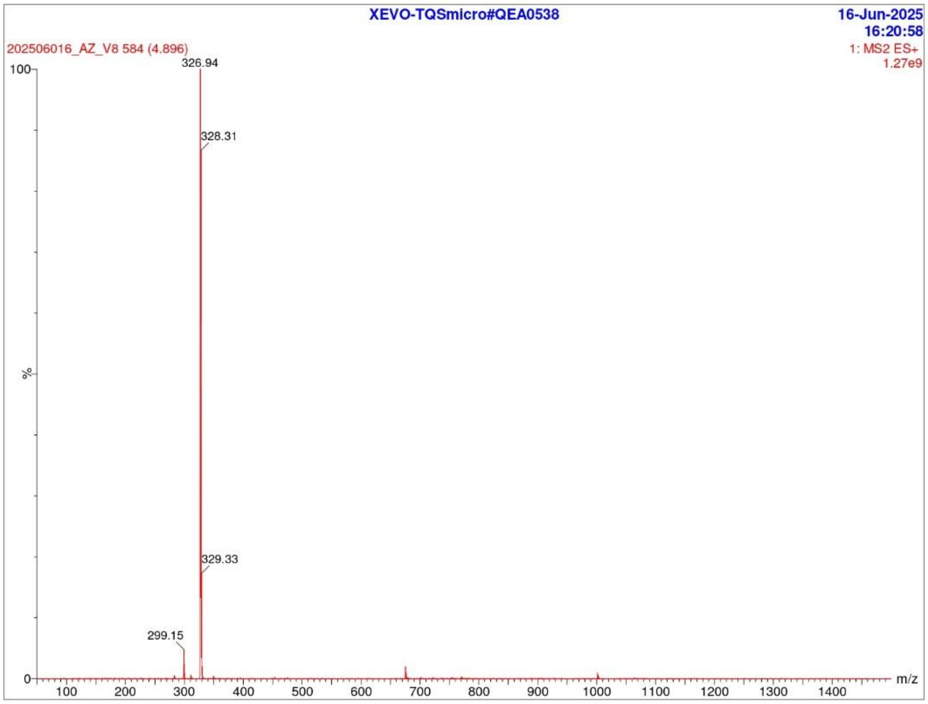

Figure S17: Mass spectrum of compound **V8**

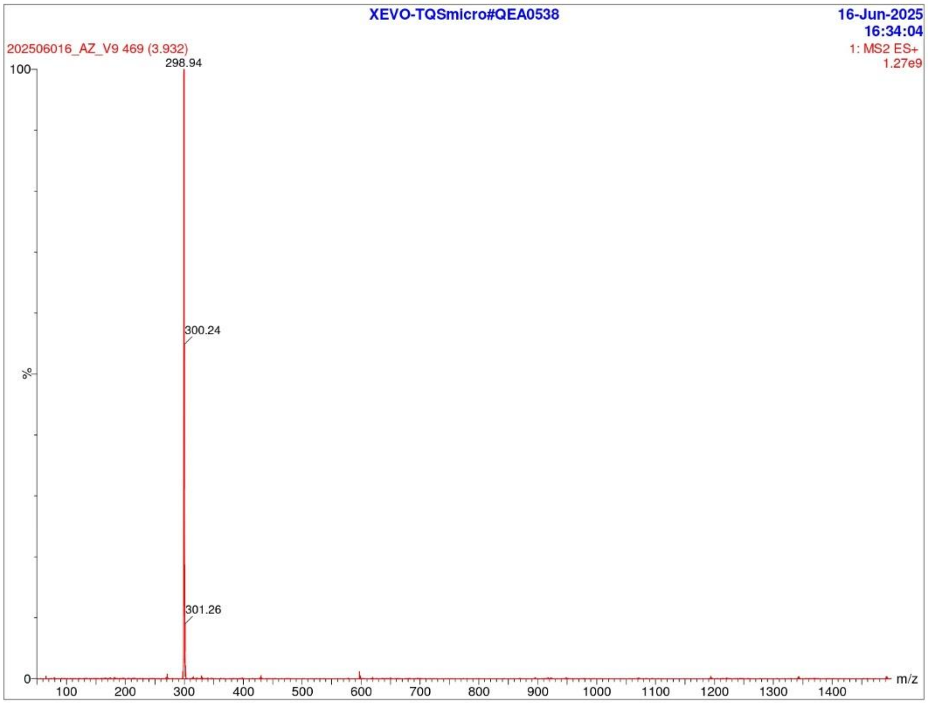

Figure S18: Mass spectrum of compound **V9**

Supplementary ^{1}HNMR data

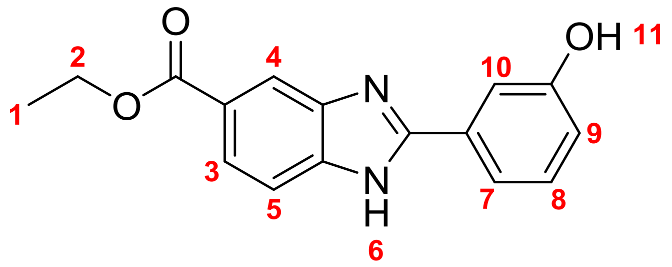

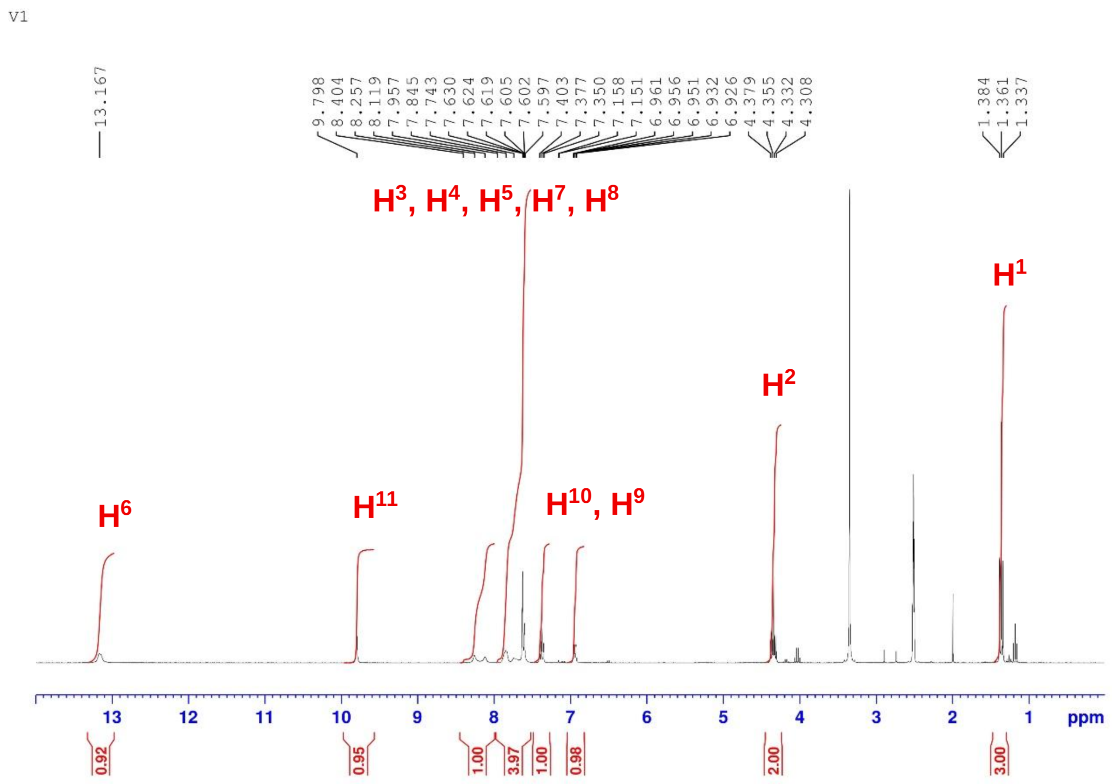

Figure S19: ^{1}H-NMR spectrum of compound **V1**

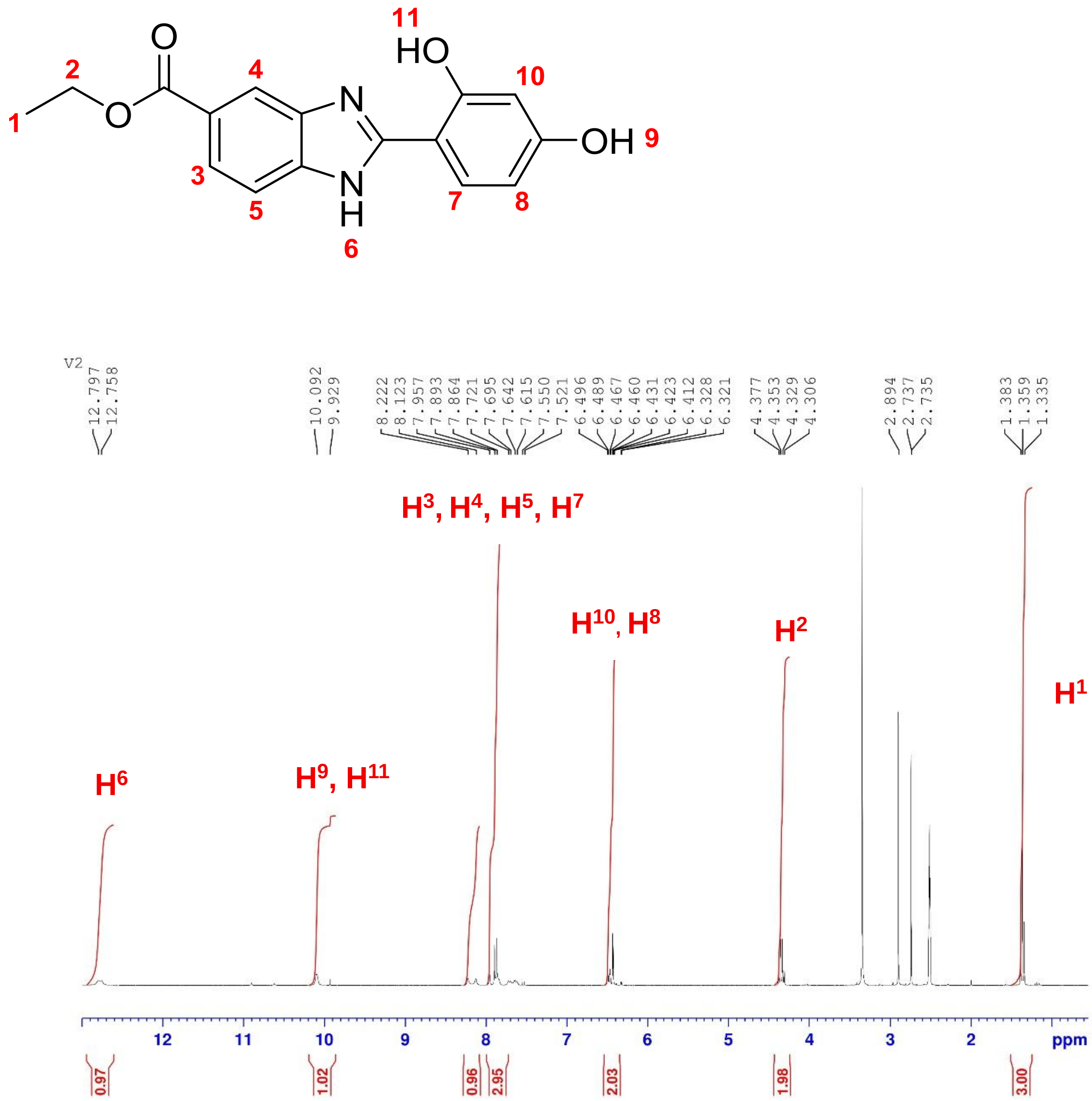

Figure S20: ^{1}H-NMR spectrum of compound **V2**

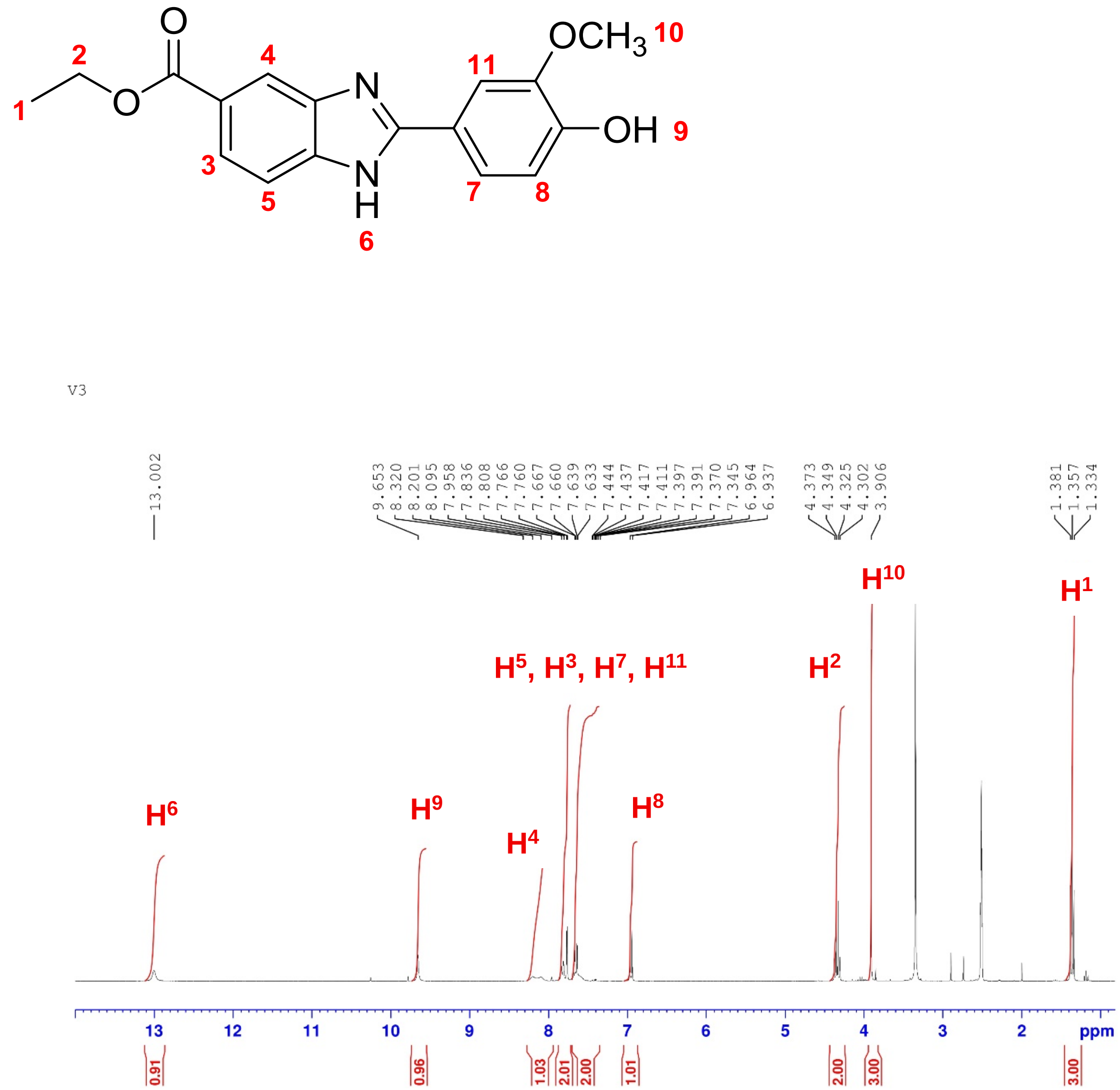

Figure S21: ^{1}H-NMR spectrum of compound **V3**

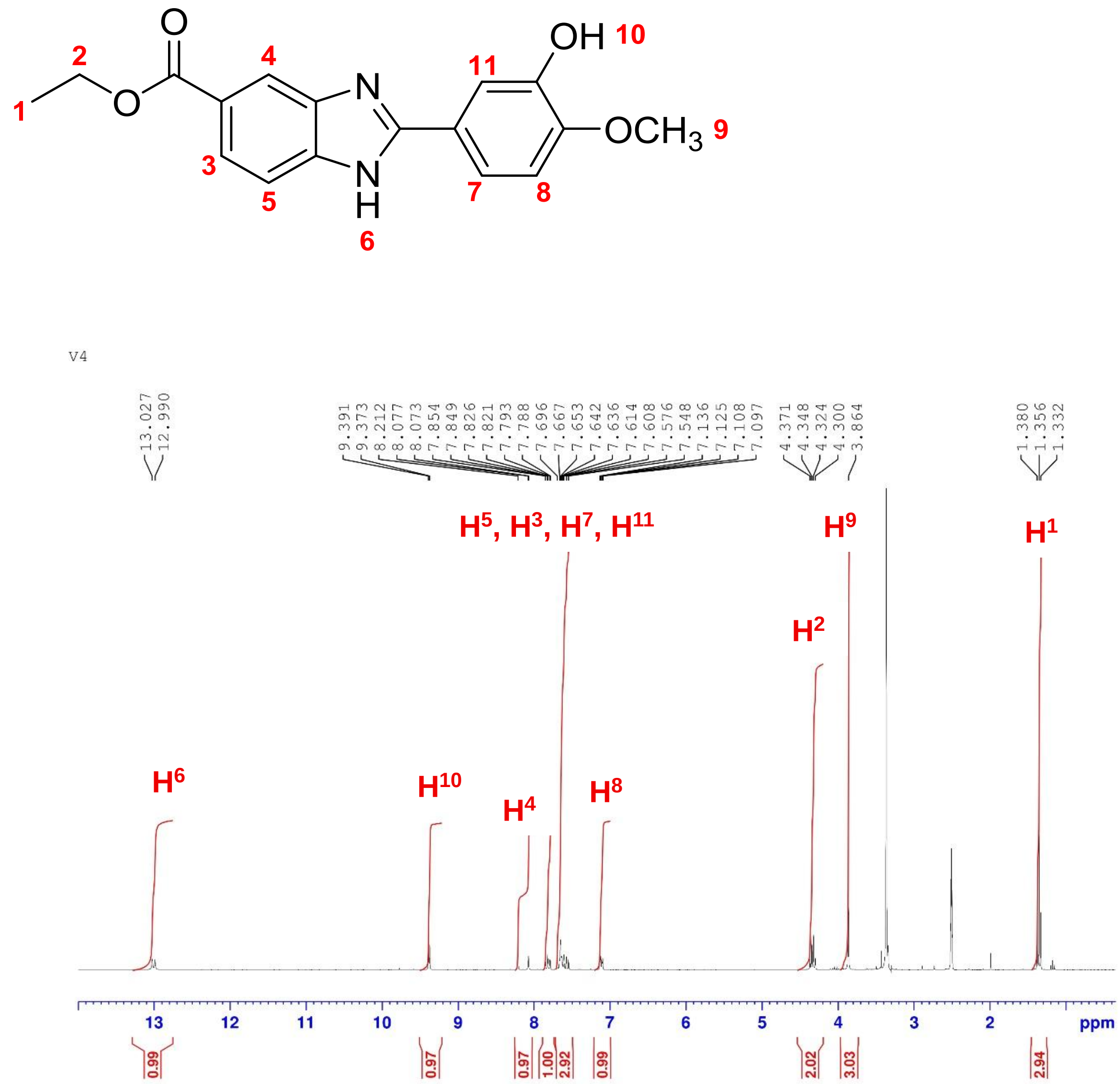

Figure S22: ^{1}H-NMR spectrum of compound **V4**

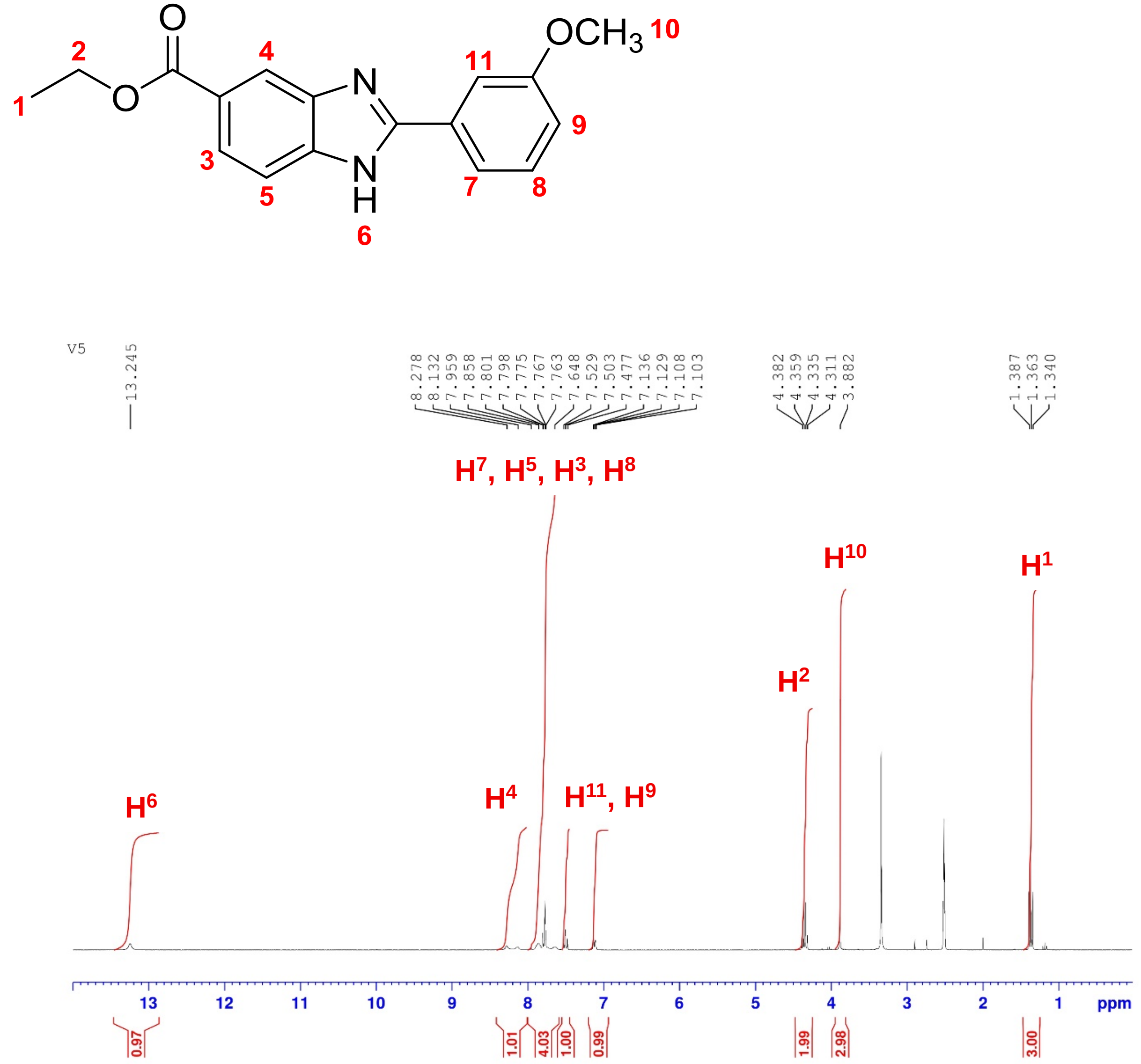

Figure S23: ^{1}H-NMR spectrum of compound **V5**

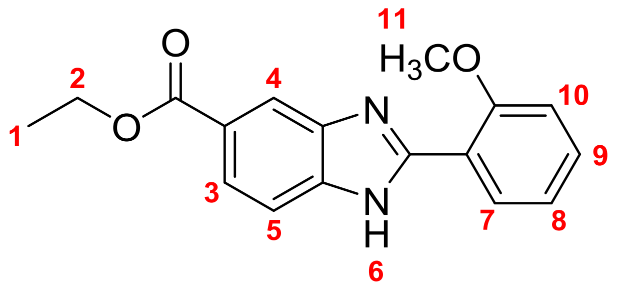

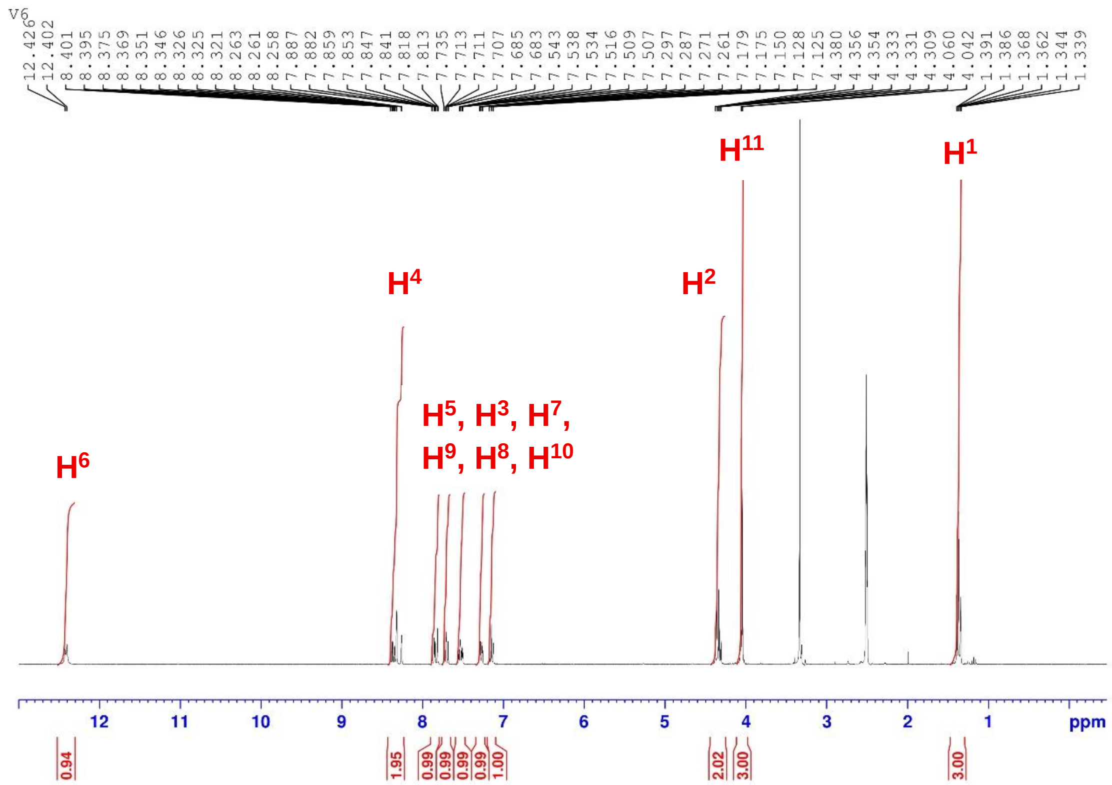

Figure S24: ^{1}H-NMR spectrum of compound **V6**

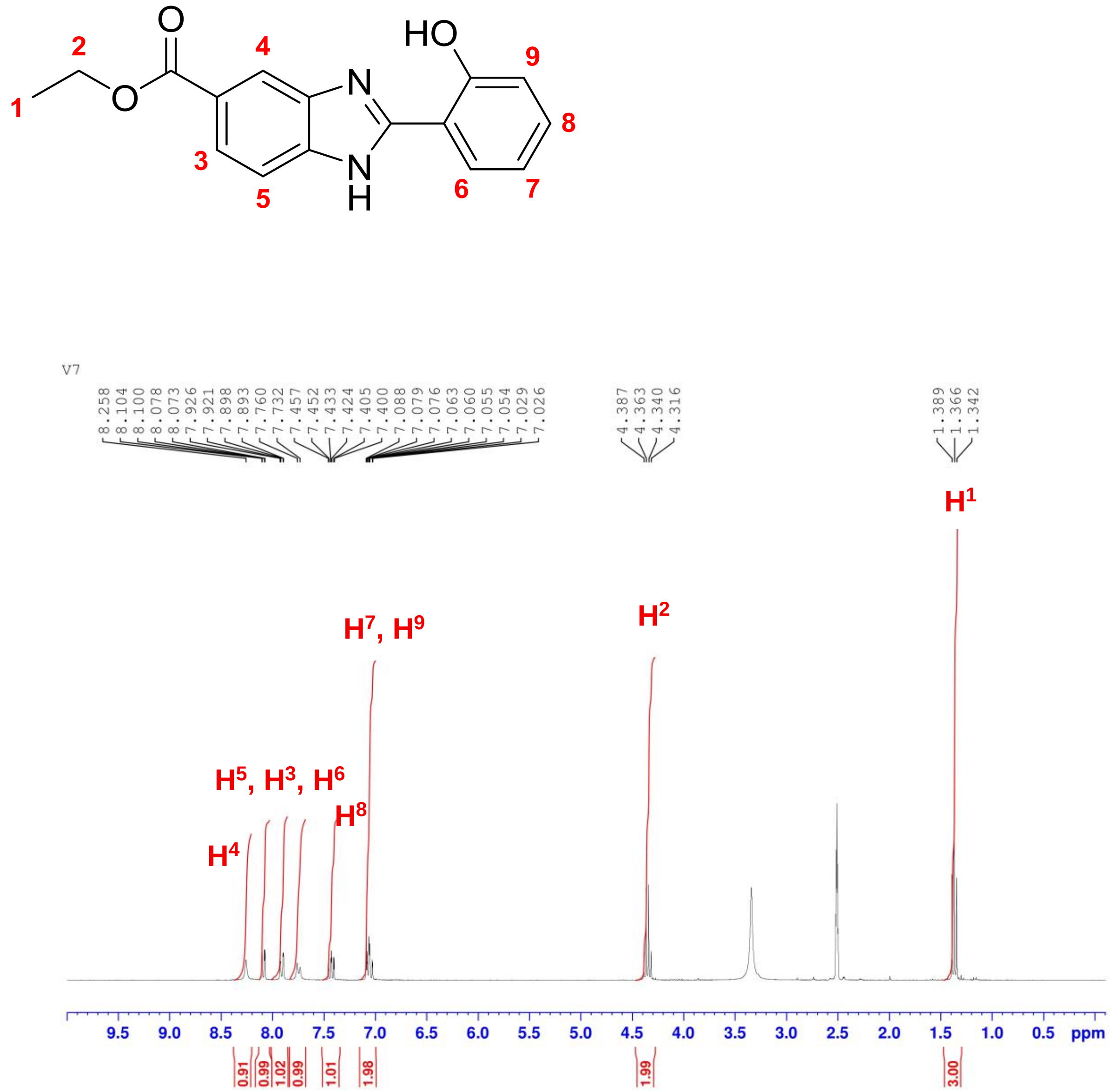

Figure S25: ^{1}H-NMR spectrum of compound **V7**

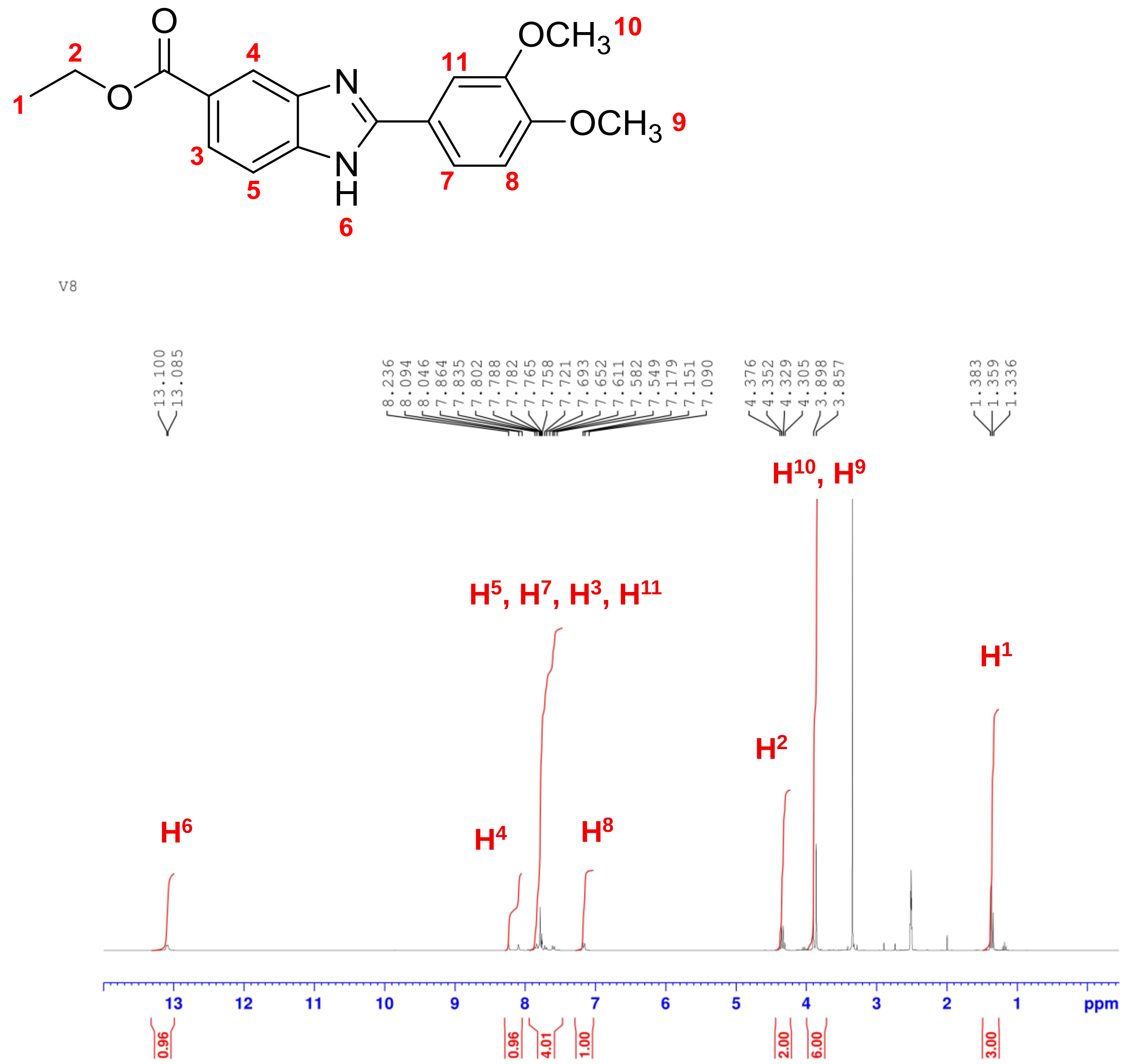

Figure S26: ^{1}H-NMR spectrum of compound **V8**

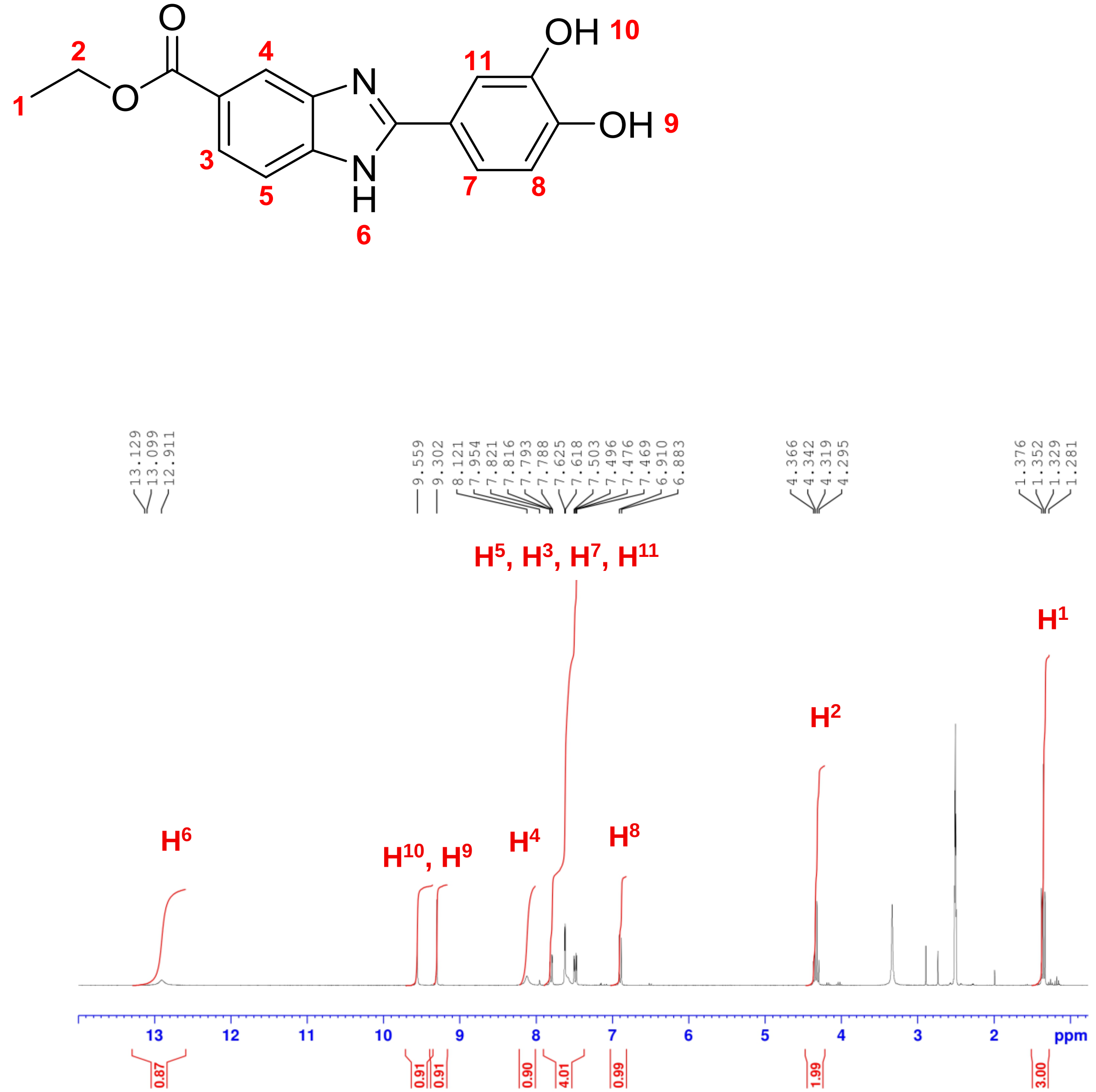

Figure S27: ^{1}H-NMR spectrum of compound **V9**

Supplementary ^{13}CNMR data

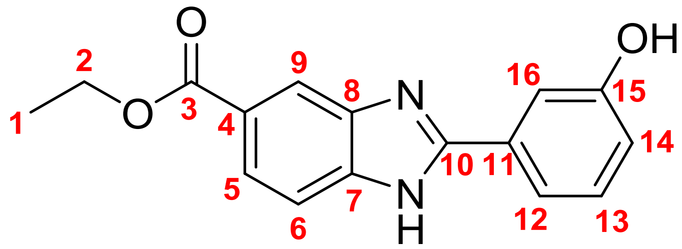

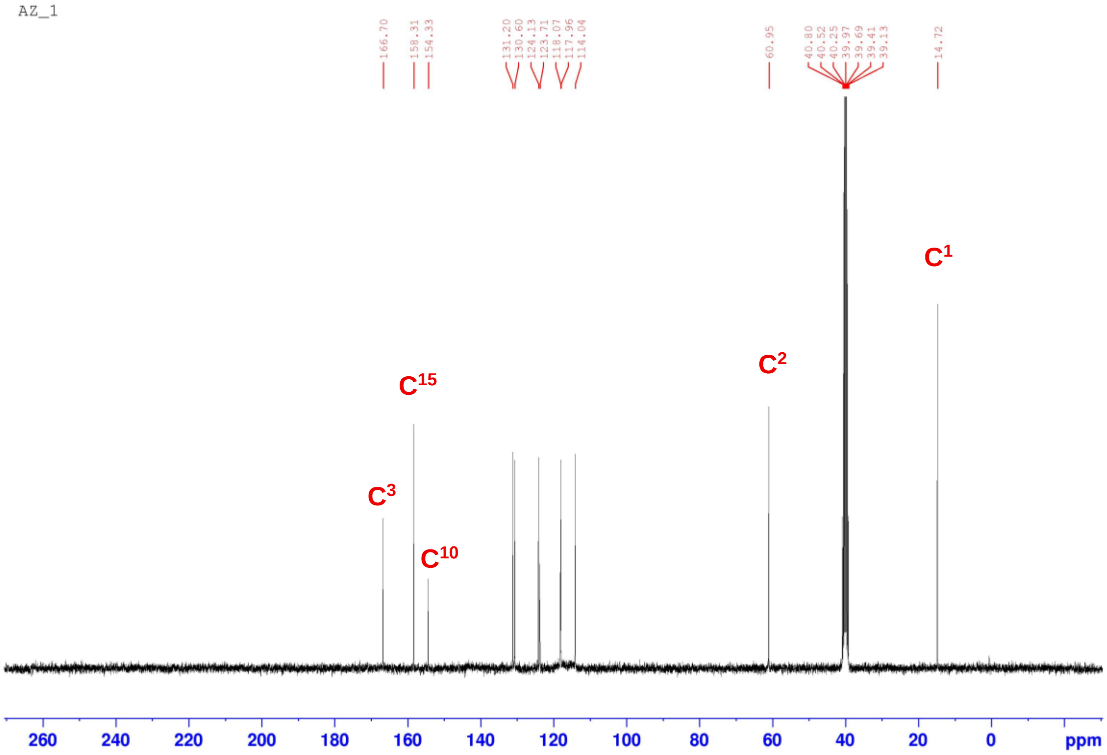

Figure S28a: ^{13}C-NMR spectrum of compound **V1**

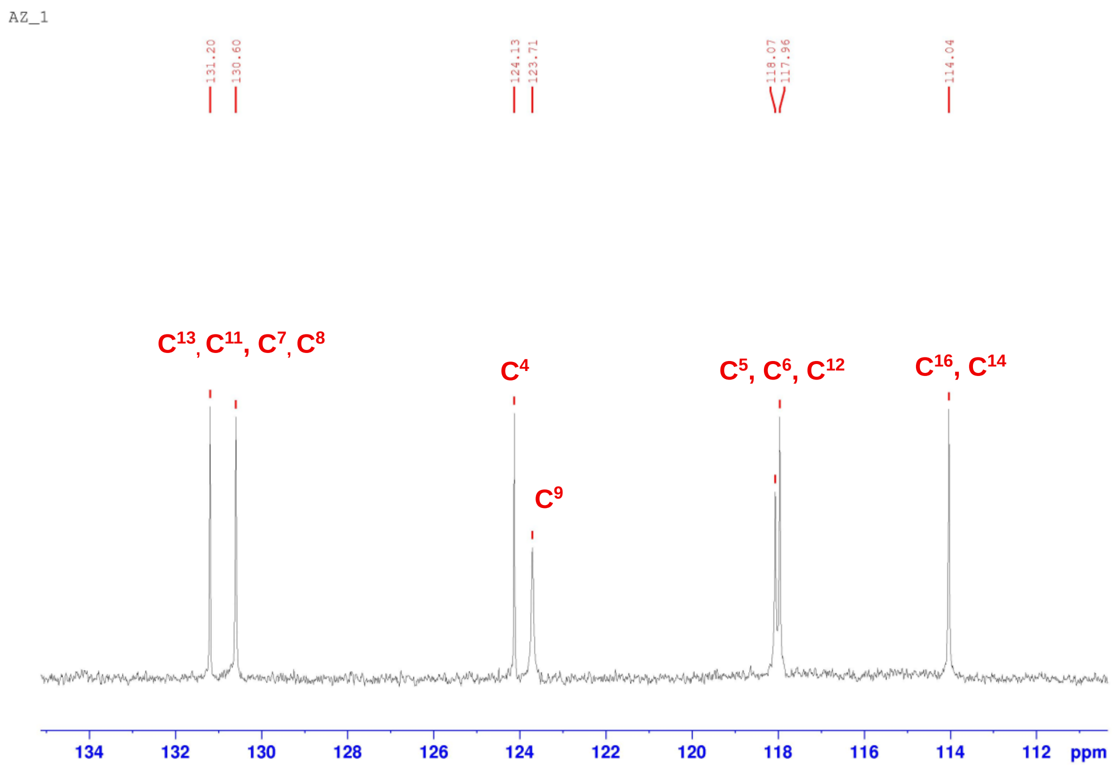

Figure S28b: ^{13}C-NMR spectrum of compound **V1**

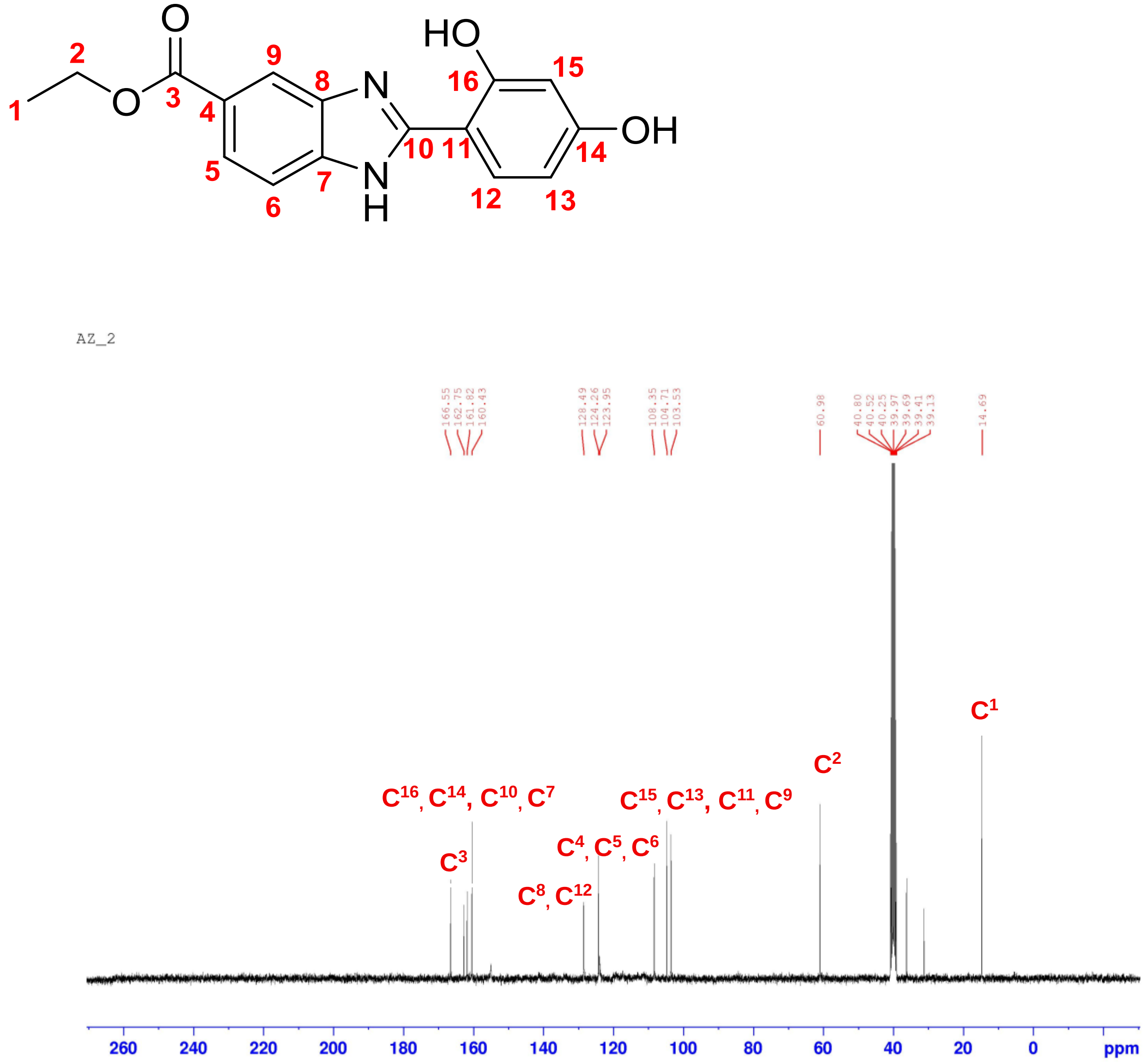

Figure S29: ^{13}C-NMR spectrum of compound **V2**

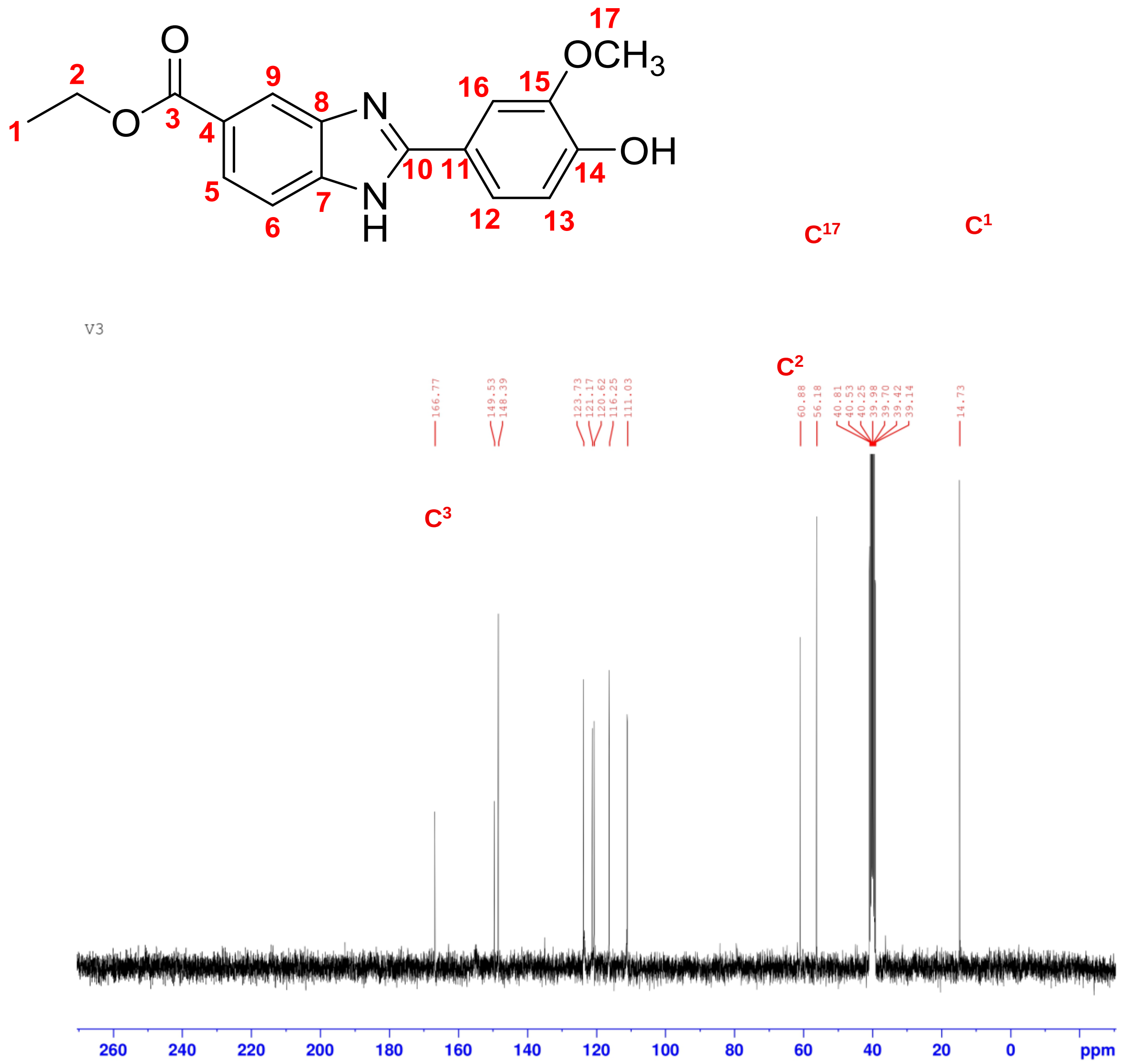

Figure S30a: ^{13}C-NMR spectrum of compound **V3**

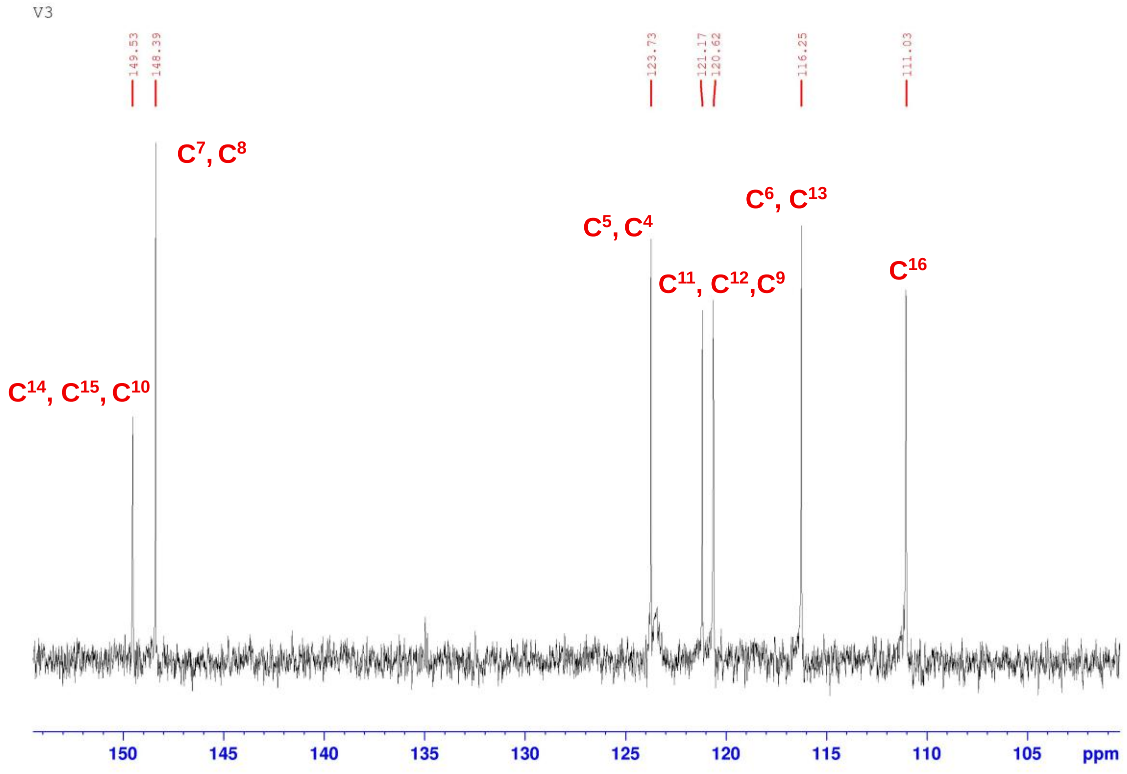

Figure S30b: ^{13}C-NMR spectrum of compound **V3**

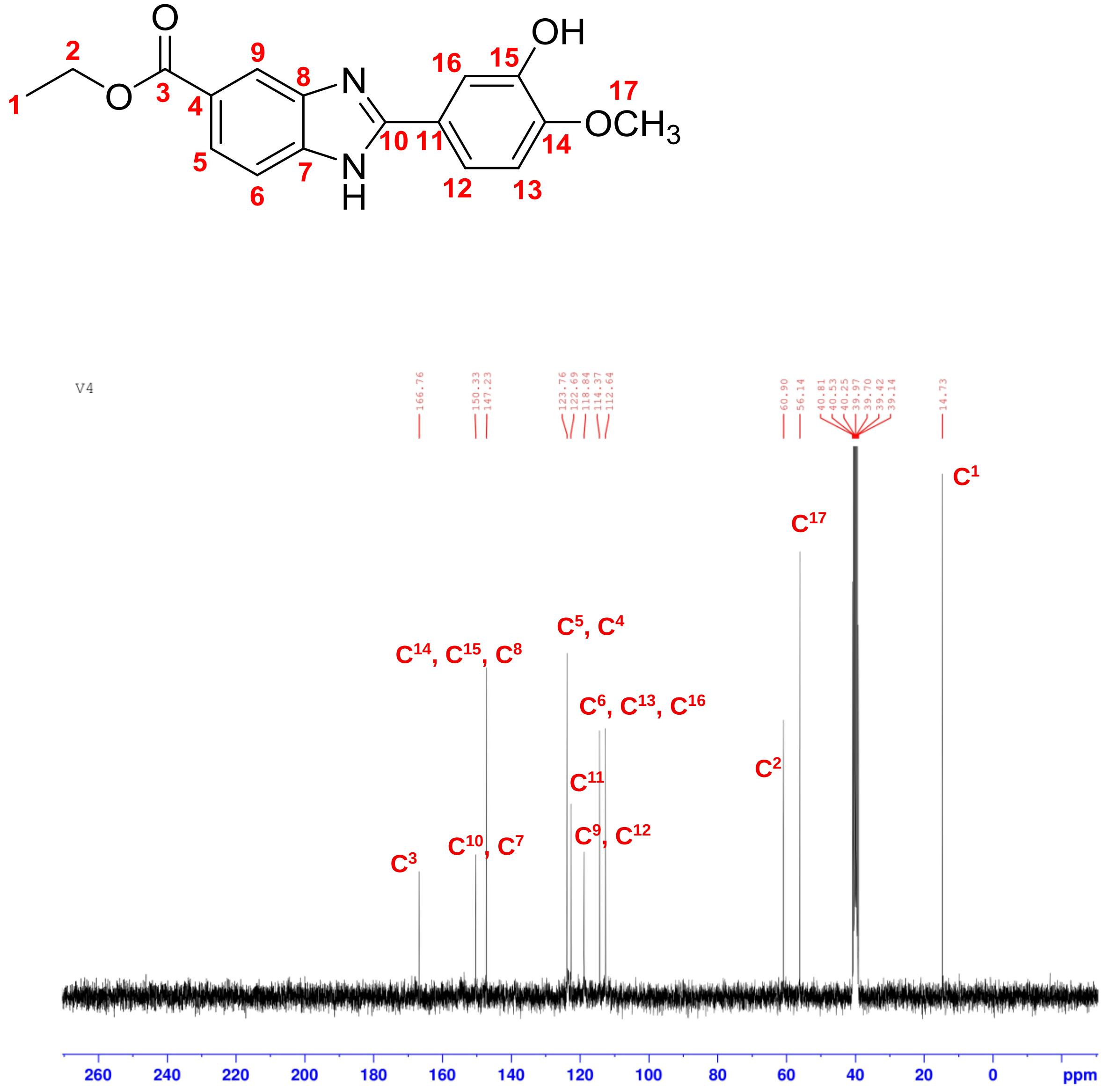

Figure S31: ^{13}C-NMR spectrum of compound **V4**

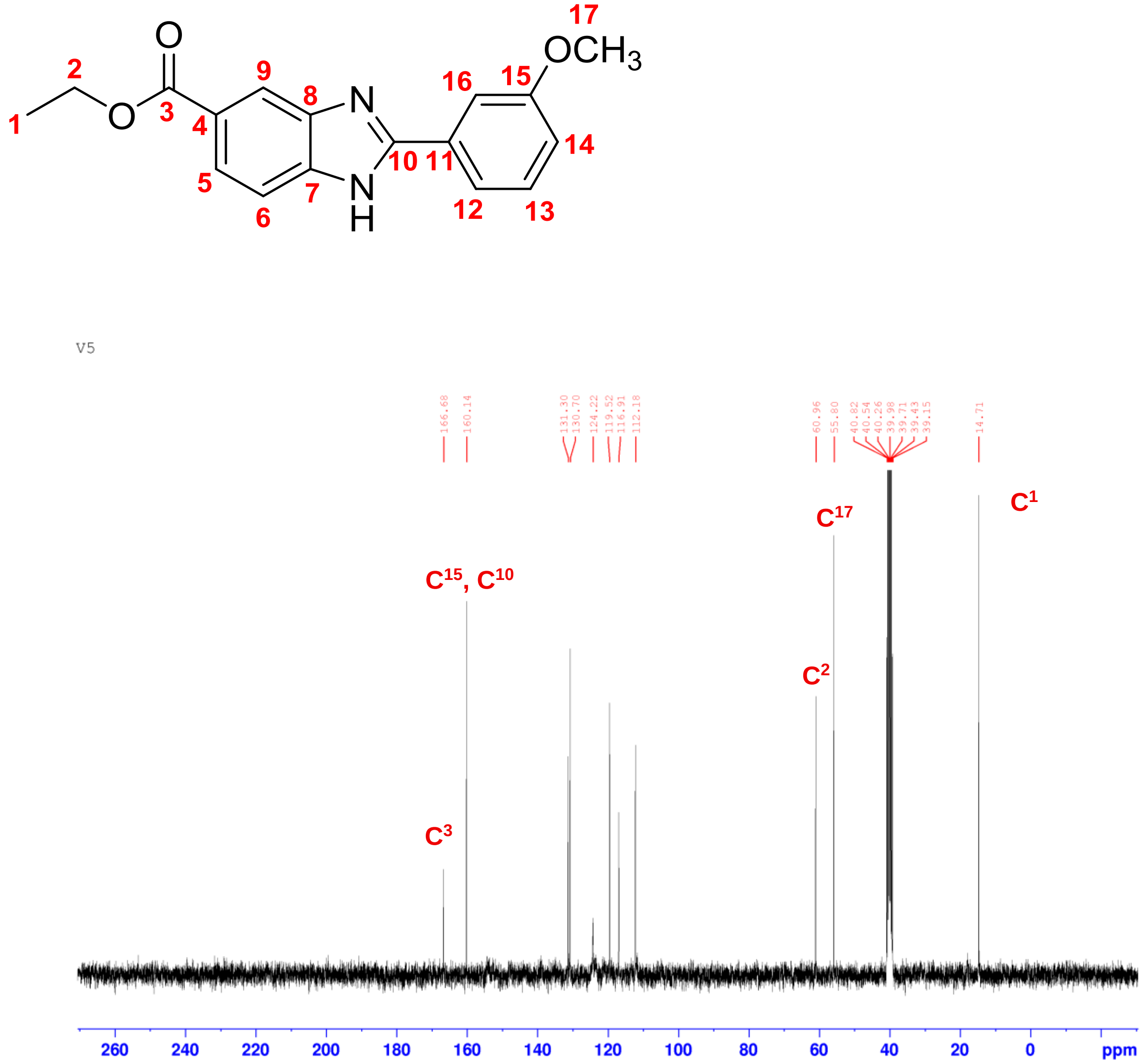

Figure S32a: ^{13}C-NMR spectrum of compound **V5**

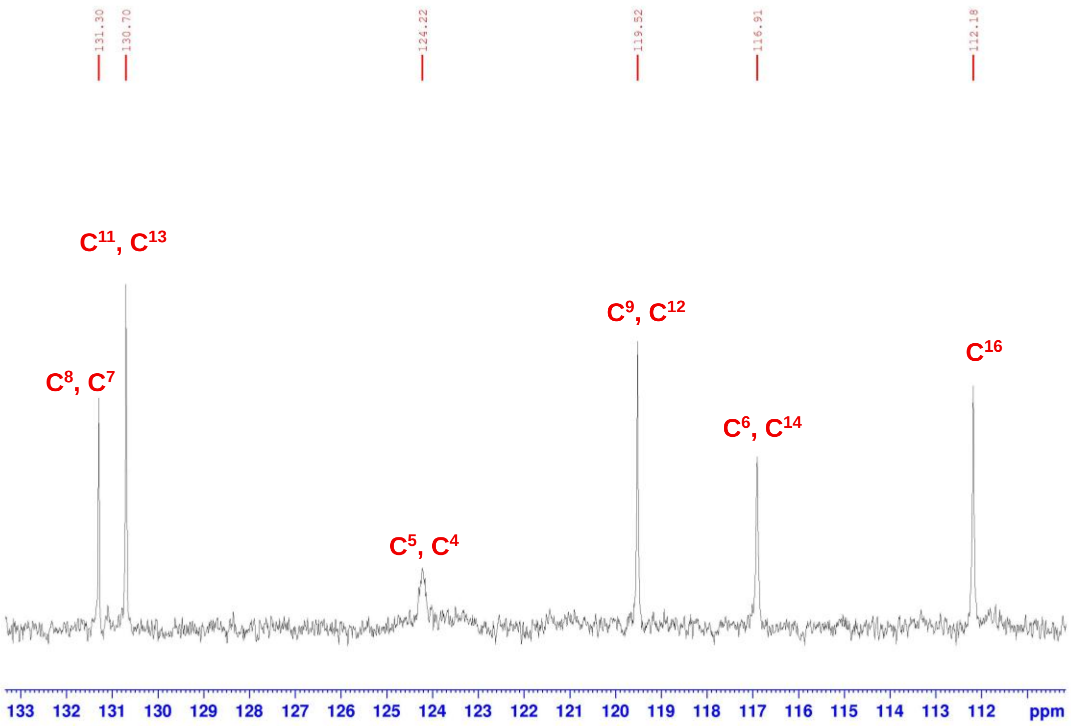

Figure S32b: ^{13}C-NMR spectrum of compound **V5**

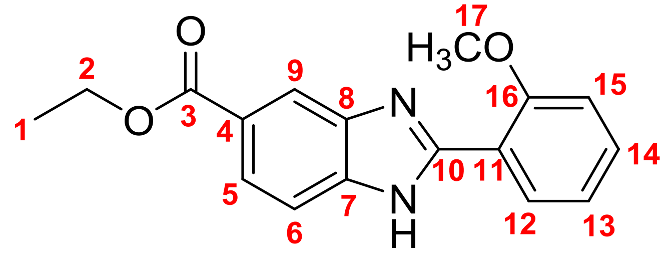

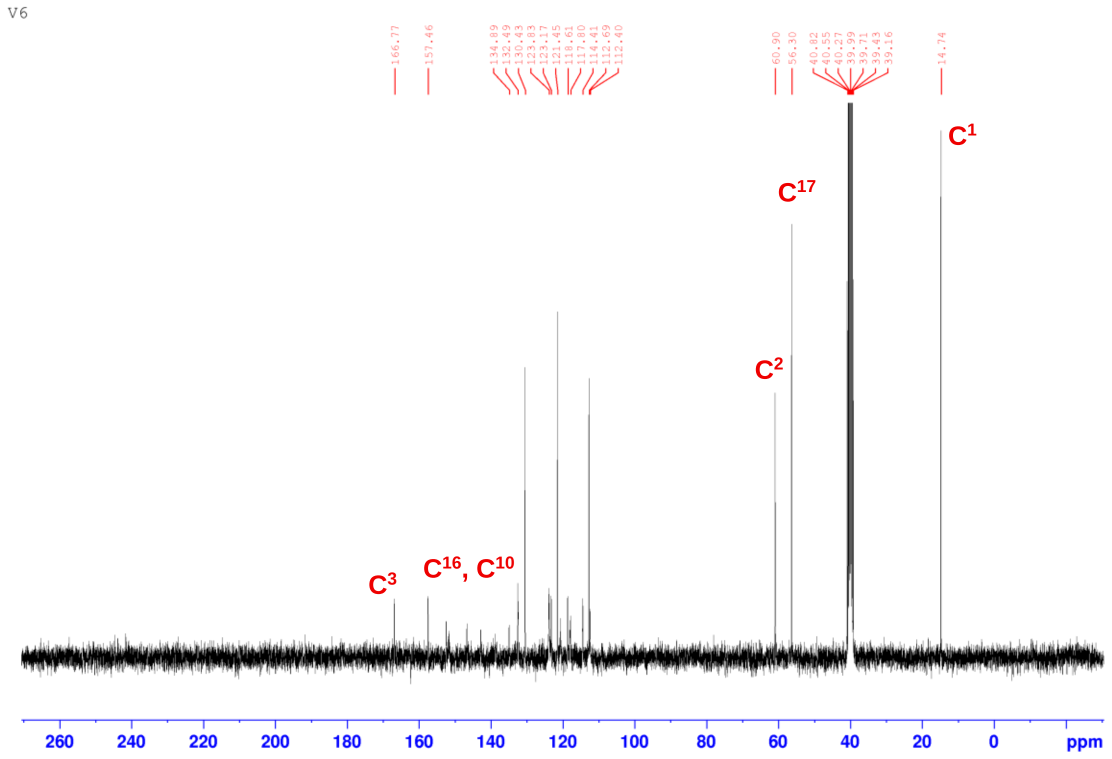

Figure S33a: ^{13}C-NMR spectrum of compound **V6**

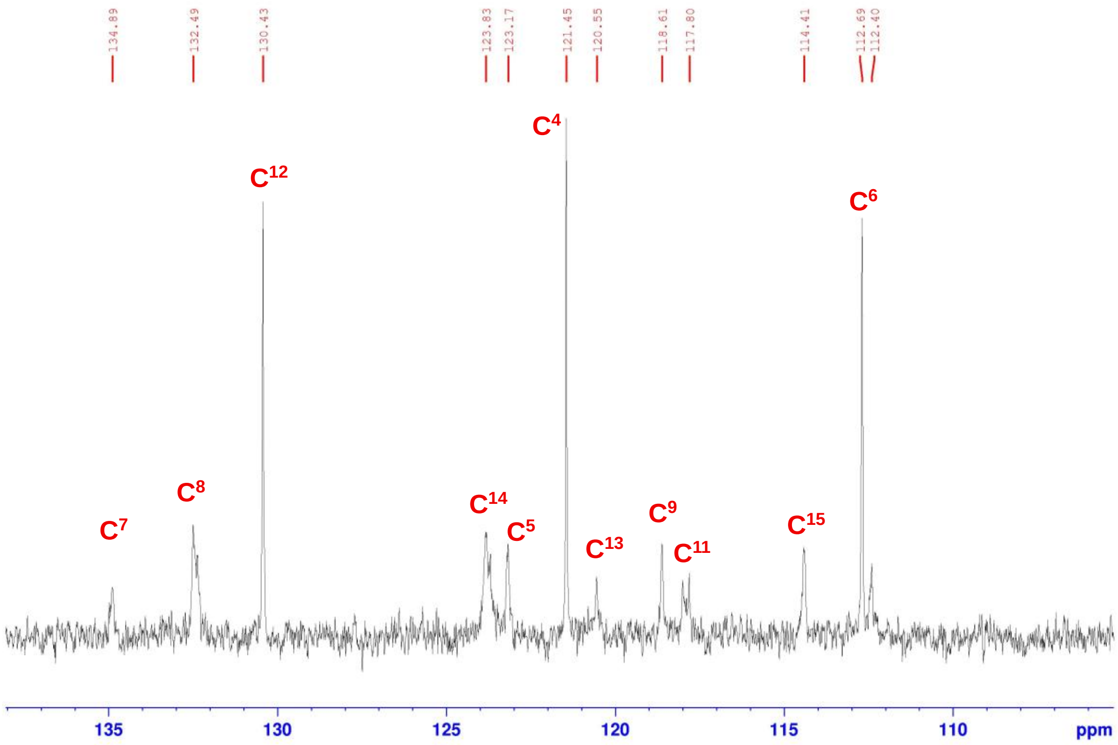

Figure S33b: ^{13}C-NMR spectrum of compound **V6**

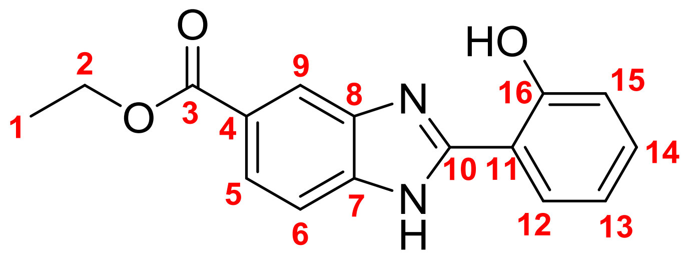

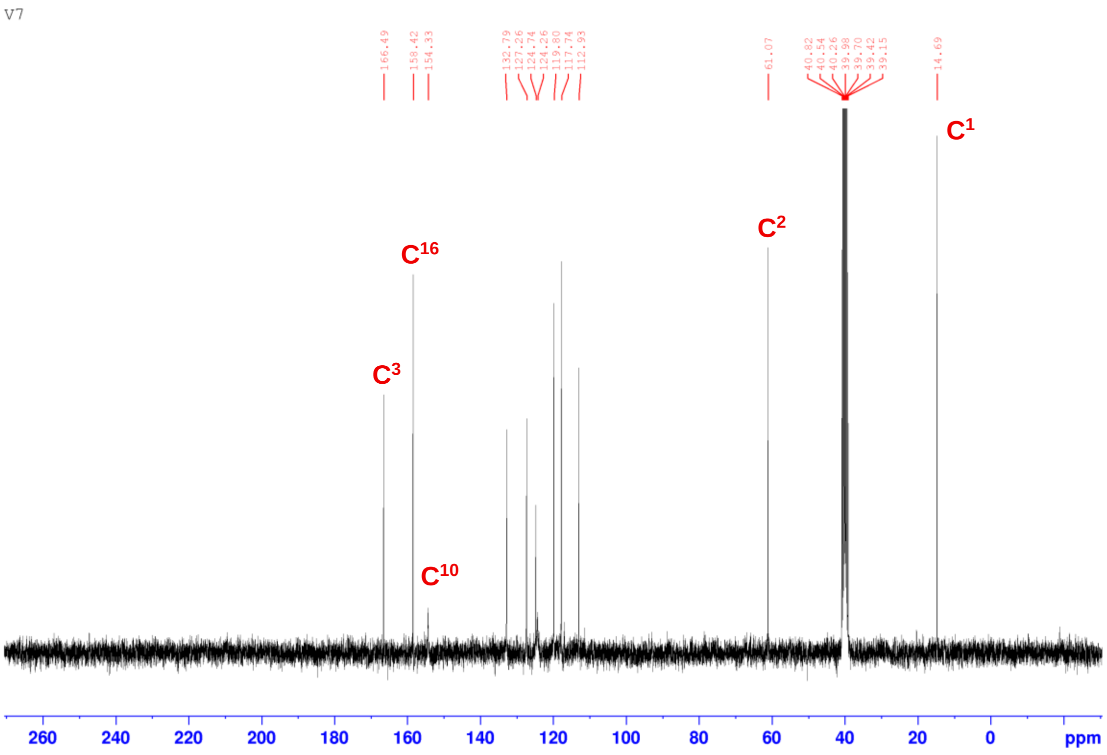

Figure S34a: ^{13}C-NMR spectrum of compound **V7**

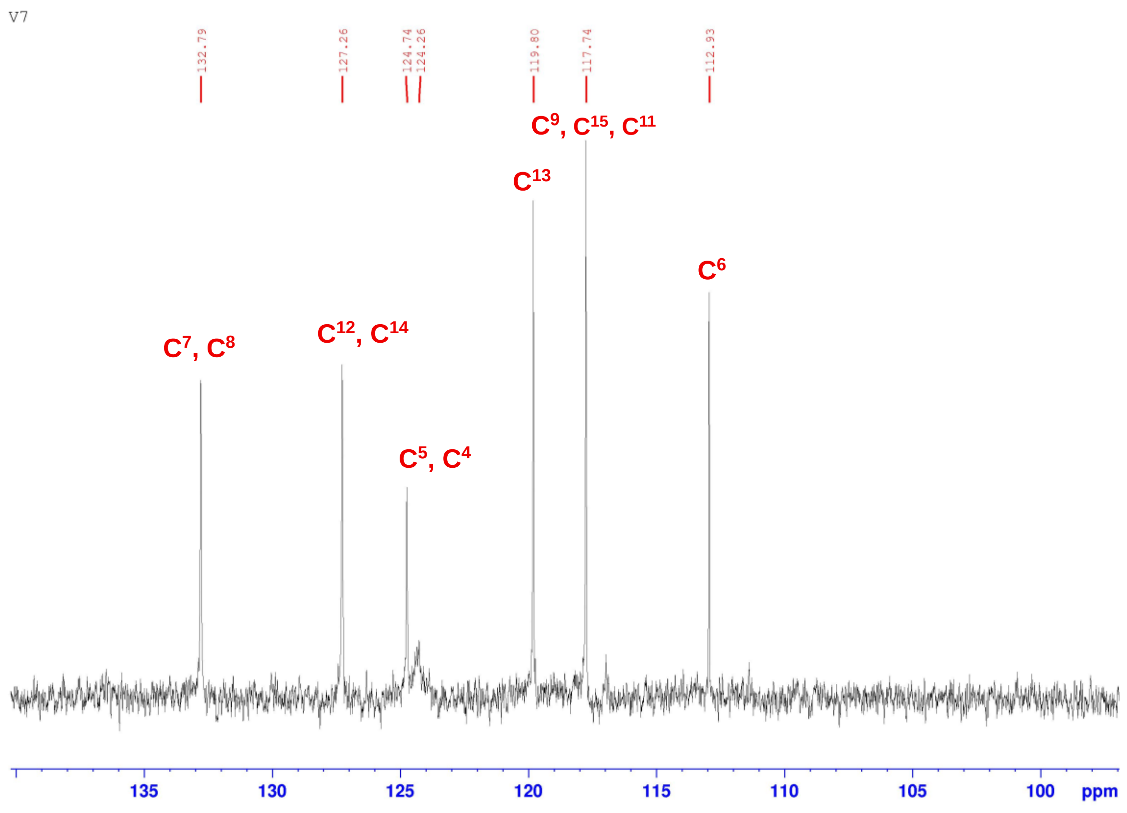

Figure S34b: ^{13}C-NMR spectrum of compound **V7**

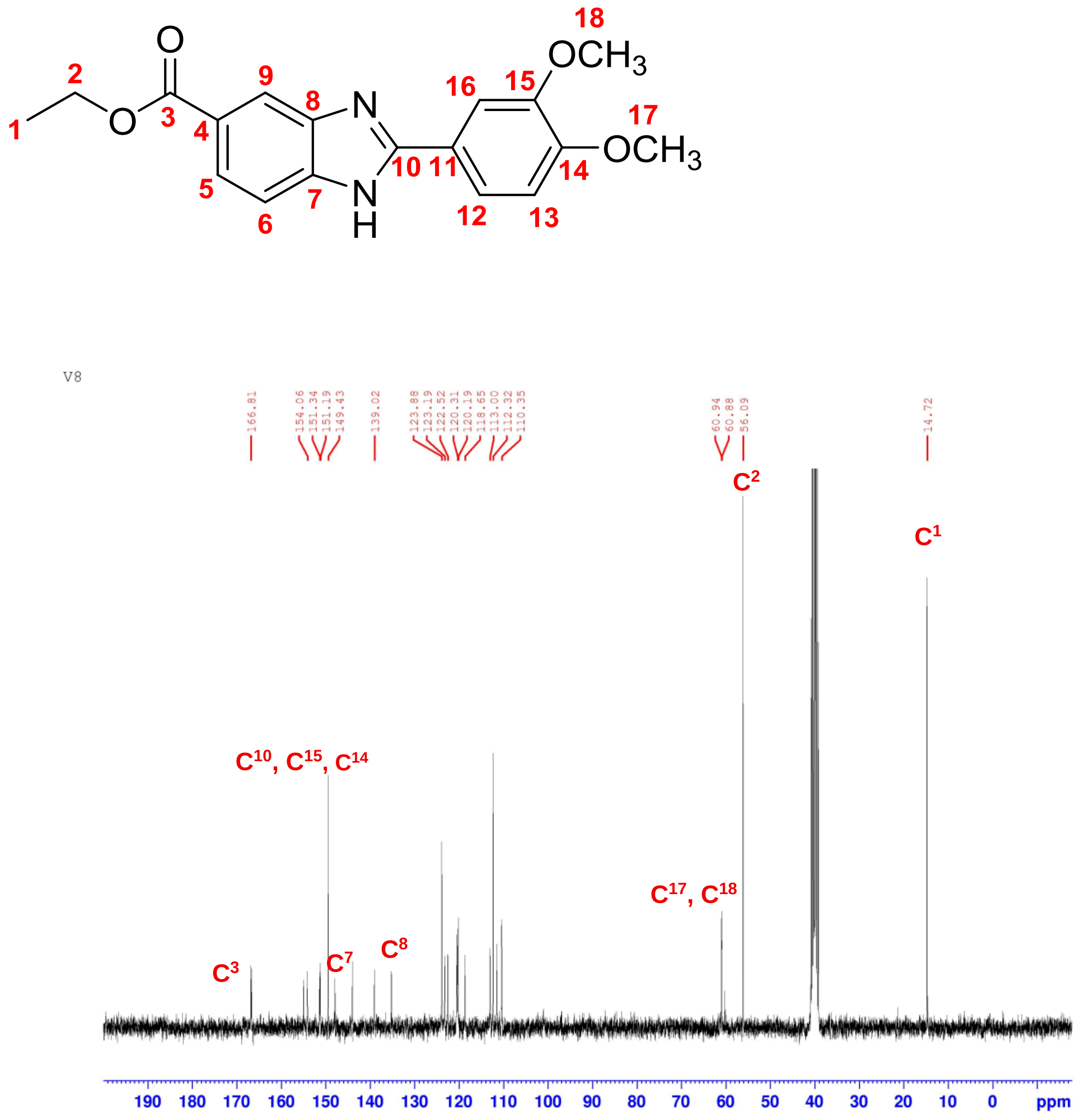

Figure S35a: ^{13}C-NMR spectrum of compound **V8**

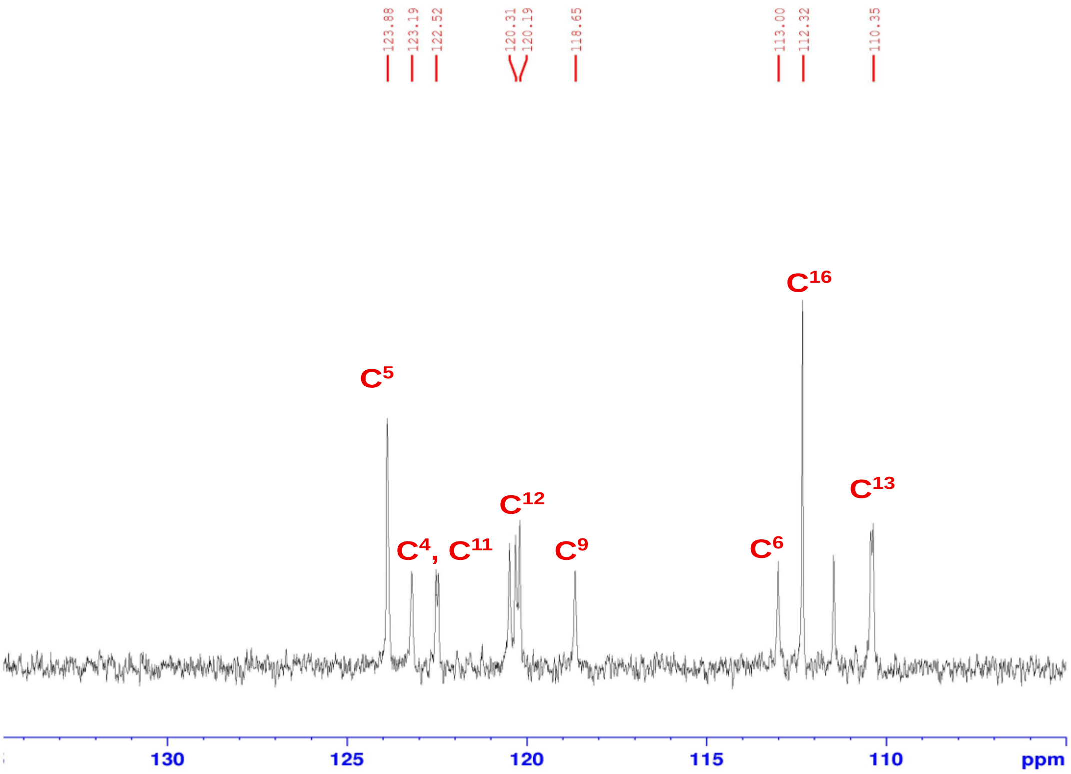

Figure S35b: ^{13}C-NMR spectrum of compound **V8**

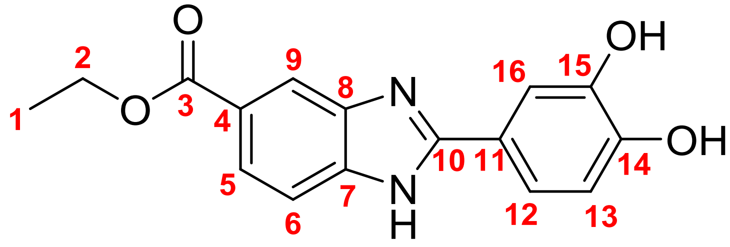

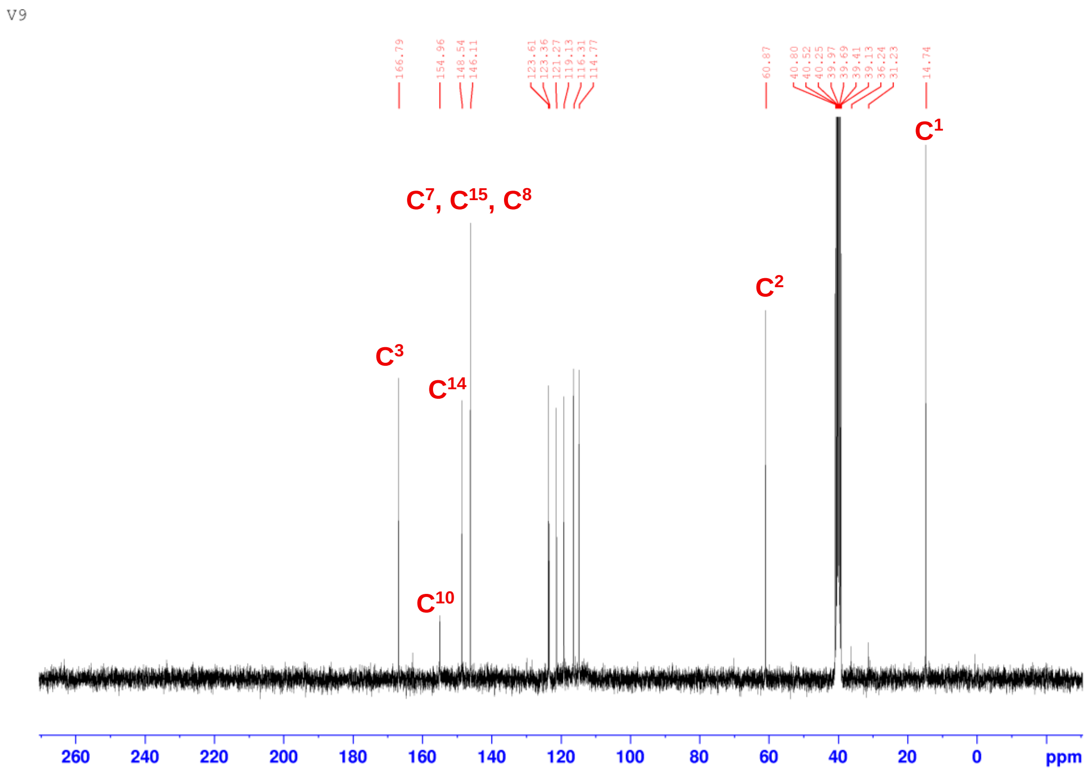

Figure S36a: ^{13}C-NMR spectrum of compound **V9**

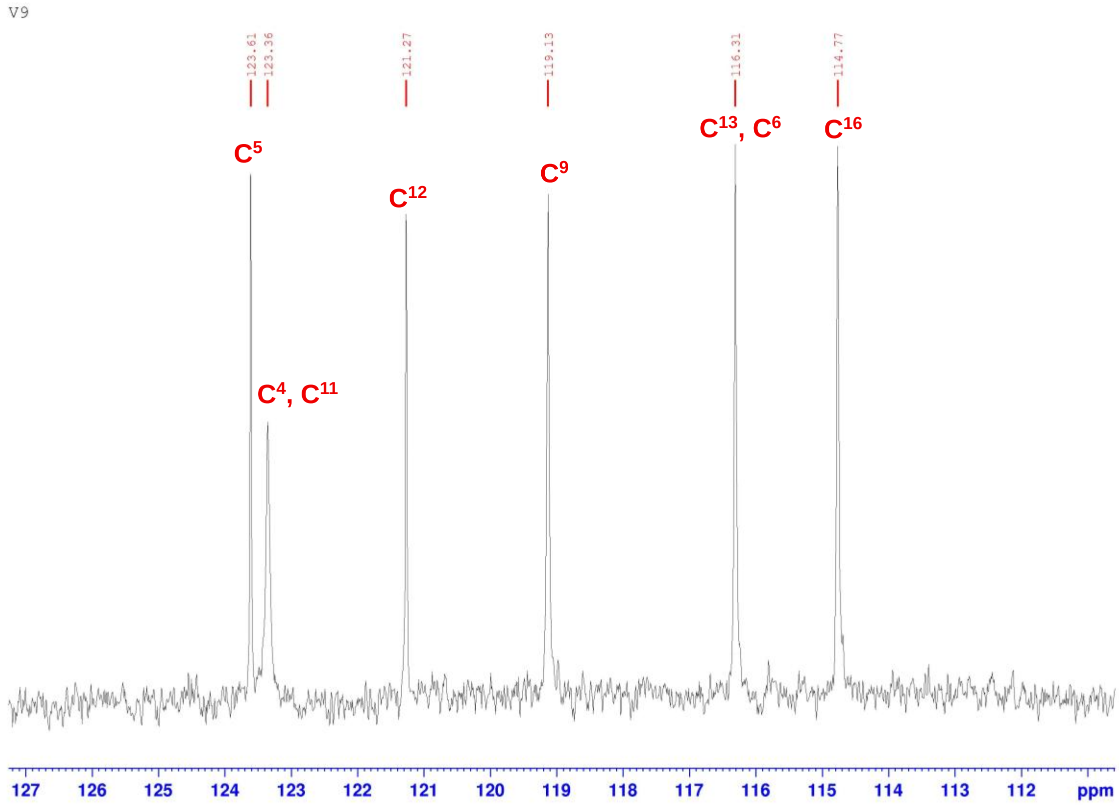

Figure S36b: ^{13}C-NMR spectrum of compound **V9**

Supplementary SIRT2 inhibition data

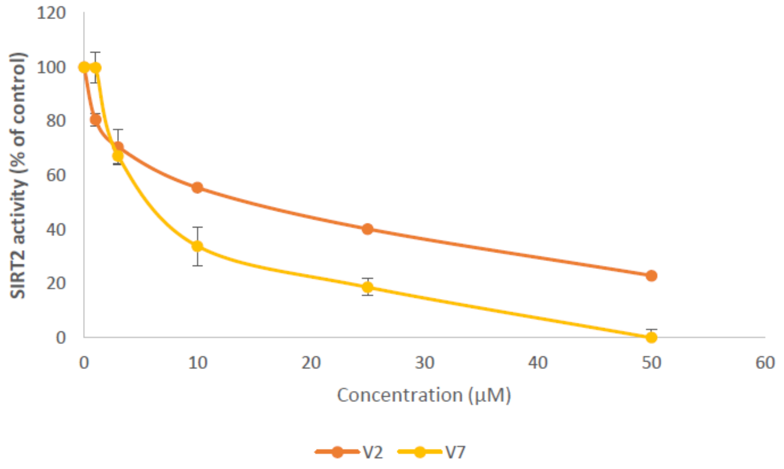

Figure S37: Dose-response curves illustrating the effect of selected compounds **V2** and **V7** on SIRT2 activity. The activity was evaluated against five different concentrations of the compounds (50 µM, 25 µM, 10 µM, 3 µM, 1 µM) using the SIRT2 Direct Fluorescent Screening Assay Kit (Cayman Chemicals). Error bars represent the Standard Error of Mean (SEM).

Table 1: Percentage inhibition at different concentrations and IC_{50} value of compounds **V2** and **V7**

Compound	Compound concentrations (µM)	% Inhibition	IC_{50} (µM)
**V2**	50	77.0 ± 0.1	10.8 ± 1.1
	25	59.8 ± 0.1	
	10	44.5 ± 0.2	
	3	29.5 ± 6.6	
	1	19.4 ± 2.4	
**V7**	50	100.0 ± 3.0	6.4 ± 0.5
	25	81.3 ± 3.2	
	10	66.1 ± 7.1	
	3	32.8 ± 2.8	
	1	0.2 ± 5.8	

Supplementary fluorescence studies data

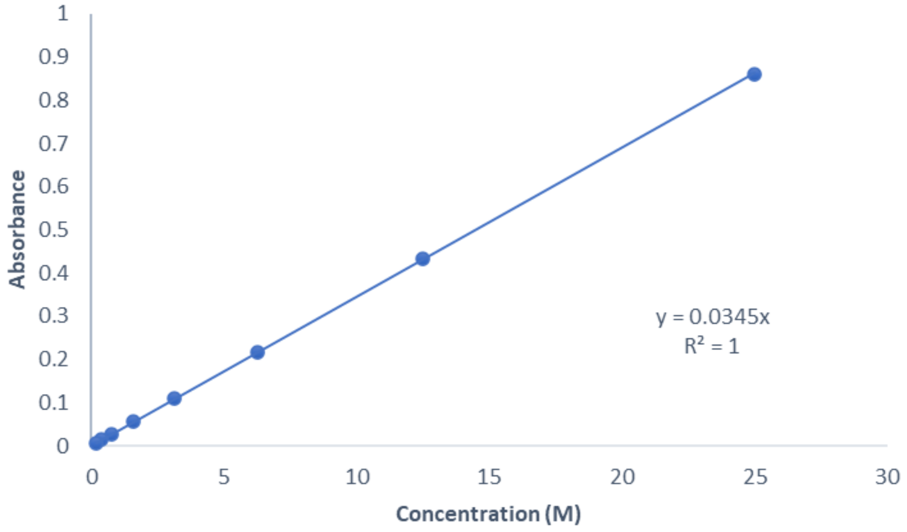

Figure S38: Plot of absorbance (326 nm) versus concentration of **V7** in DMSO. Molar absorptivity (molar extinction coefficient, ε) was determined by the gradient of the slope using Beer Lamberts equation. Absorbance readings were taken in a quartz cuvette (10 mm path length), using UviLine 9400 spectrophotometer. The excitation wavelength was set at 326 nm.

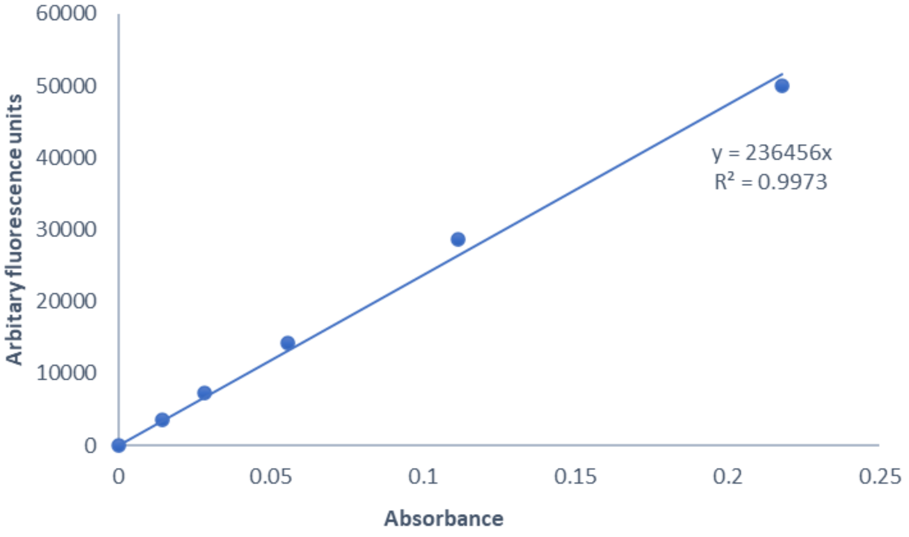

Figure S39: Plot of fluorescence intensity versus absorbance for **V7** in DMSO. Arbitrary fluorescence values were determined using the Tecan Infinite 200 Pro, set at an excitation wavelength of 326 nm and an emission wavelength of 492 nm. Absorbance readings were taken in a quartz cuvette (10 mm path length), using the UviLine 9400 spectrophotometer.

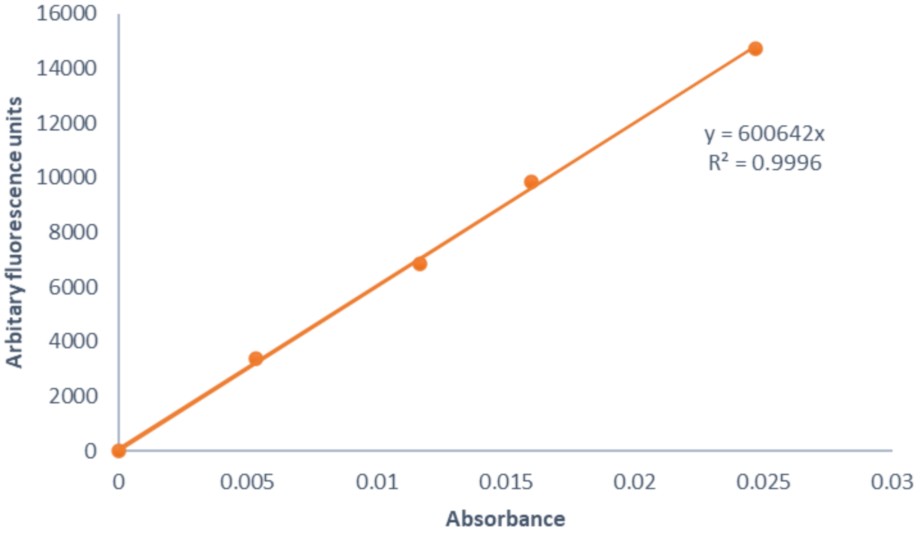

Figure S40: Plot of fluorescence intensity versus absorbance for the quinine sulphate reference in 0.5 M H_2SO_4. Arbitrary fluorescence values were determined using the

Tecan Infinite 200 Pro, set at an excitation wavelength of 348 nm and an emission wavelength of 450 nm. Absorbance readings were taken in a quartz cuvette (10 mm path length), using the UviLine 9400 spectrophotometer.
